# The Diversity of Mammalian Hemoproteins and Microbial Heme Scavengers Is Shaped by an Arms Race for Iron Piracy

**DOI:** 10.3389/fimmu.2018.02086

**Published:** 2018-09-11

**Authors:** Alessandra Mozzi, Diego Forni, Mario Clerici, Rachele Cagliani, Manuela Sironi

**Affiliations:** ^1^Scientific Institute, IRCCS E. Medea, Bioinformatics, Lecco, Italy; ^2^Department of Physiopathology and Transplantation, University of Milan, Milan, Italy; ^3^Don C. Gnocchi Foundation ONLUS, IRCCS, Milan, Italy

**Keywords:** iron piracy, nutritional immunity, positive selection, hemoglobin, hemopexin

## Abstract

Iron is an essential micronutrient for most living species. In mammals, hemoglobin (Hb) stores more than two thirds of the body's iron content. In the bloodstream, haptoglobin (Hp) and hemopexin (Hpx) sequester free Hb or heme. Pathogenic microorganisms usually acquire iron from their hosts and have evolved complex systems of iron piracy to circumvent nutritional immunity. Herein, we performed an evolutionary analysis of genes coding for mammalian heme-binding proteins and heme-scavengers in pathogen species. The underlying hypothesis is that these molecules are engaged in a molecular arms race. We show that positive selection drove the evolution of mammalian Hb and Hpx. Positively selected sites in Hb are located at the interaction surface with *Neisseria meningitidis* heme scavenger HpuA and with *Staphylococcus aureus* iron-regulated surface determinant B (IsdB). In turn, positively selected sites in HpuA and IsdB are located in the flexible protein regions that contact Hb. A residue in Hb (S45H) was also selected on the Caprinae branch. This site stabilizes the interaction with *Trypanosoma brucei* hemoglobin-haptoglobin (HbHp) receptor (*Tb*HpHbR), a molecule that also mediates trypanosome lytic factor (TLF) entry. In *Tb*HpHbR, positive selection drove the evolution of a variant (L210S) which allows evasion from TLF but reduces affinity for HbHp. Finally, selected sites in Hpx are located at the interaction surface with the *Haemophilus influenzae* hemophore HxuA, which in turn displays fast evolving sites at the Hpx-binding interface. These results shed light into host-pathogens conflicts and establish the importance of nutritional immunity as an evolutionary force.

## Introduction

Iron is an essential micronutrient and serves as an ideal redox catalyst for basic cellular processes including respiration and oxygen transport. However, this redox potential contributes to its high toxicity (1). In humans, as in the majority of vertebrates, iron distribution is finely controlled. About two thirds of total iron in the body is complexed within the porphyrin ring of heme as a cofactor of hemoglobin (Hb) or myoglobin. At the intracellular level, ferritin also contributes to iron storage and regulates its availability in the cell. Iron that is released upon cell lysis is quickly sequestered by specific proteins, such as transferrin, albumin, lactoferrin, and hemopexin to prevent oxidative damage (2, 3). These proteins have very high binding affinity for free and/or heme-complexed iron (2).

Pathogenic bacteria and parasites also depend on iron for their metabolic processes and usually acquire this metal from their hosts. Thus, iron sequestration by host proteins prevents toxicity and simultaneously limits its availability to invading microbes, a situation referred to as “nutritional immunity” (4). As a consequence of this, pathogens have developed a plethora of molecular mechanisms to circumvent nutritional immunity in order to scavenge iron from host proteins (5). Bacterial pathogens, in particular, display very diversified molecular strategies of iron piracy (5–7). Eukaryotic parasites such as *Trypanosoma brucei*, the causative agent of African sleeping sickness, also target host iron-binding proteins (e.g., transferrin and hemoglobin-haptoglobin complexes) for iron acquisition (8–11).

The competition for iron can thus be regarded as a molecular arms-race between host iron-binding proteins and microbial iron-scavengers (6). Molecular arms races often develop into genetic conflicts whereby cyclical adaptation and counteradaptation occur both in the host and in the pathogen genomes (12). Indeed, previous work indicated that the iron transport protein transferrin in great apes and TbpA, a transferrin surface receptor expressed by several pathogenic bacteria, have been engaged in an evolutionary conflict (13). Both interactors show signatures of positive selection, which are mainly localized at sites within the binding interface (13). These findings raise the question as to whether other proteins involved in nutritional immunity are similarly involved in molecular arms races (6). This is highly likely, as heme-binding proteins (hemoproteins) such as Hb and hemopexin (Hpx) represent major reservoirs of iron and are targeted by several pathogens that naturally infect humans and other mammals (7, 14).

To date, a number of iron uptake systems have been characterized for different pathogenic organisms (7, 15). Herein, we aimed to assess whether mammalian heme-binding proteins and pathogen-encoded heme scavengers are engaged in molecular arms races. We thus focused our attention on microbial molecules that directly interact with heme-binding proteins (e.g., systems that scavenge free heme were not considered). Clearly, microbial molecules may evolve in response to different pressures exerted by the host, the most prominent one being the immune system. Likewise, microbial pathogens are not the sole driver of mammalian hemoprotein evolution. Therefore, we selected for our study a subset of host-pathogen interactions with known molecular details, either crystallographic or biochemical, on the protein portions/residues that directly participate in the binding. This allows inference on the underlying selective pressure: if the binding partners have been exerting a mutual selective pressure, the selected residues are expected to be mostly located at the binding interface (12).

Results of evolutionary analyses showed that mammalian hemoproteins and microbial iron acquisition systems exerted a mutual selective pressure resulting in widespread positive selection.

## Materials and methods

### Study design

The aim of our study was to determine whether mammalian heme-binding proteins have been engaged in a molecular arms race with microbial heme-acquisition systems. We thus focused on the three major heme-binding proteins, namely Hb, Hp, and Hpx. We excluded the α subunit of Hb due to the impossibility of establishing orthology among mammalian genes (16), and Hp due to extensive copy number variation in humans (17, 18). Thus, evolutionary analyses were performed for Hpx and the Hb β subunit.

**Figure 1:**
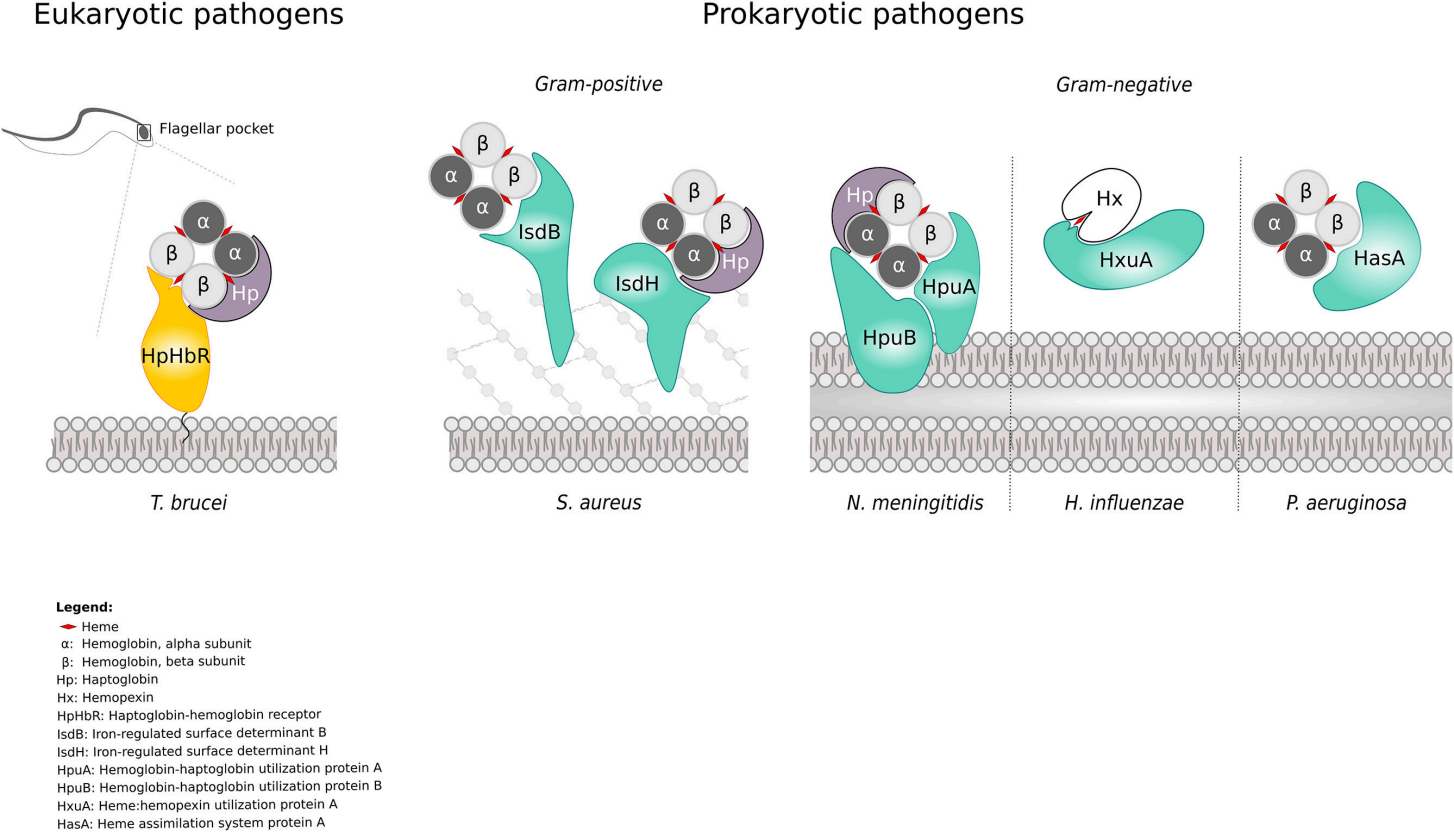
Heme uptake systems in eukaryotic and prokaryotic pathogens. Schematic overview of the analyzed interactions between host hemoproteins and pathogen-encoded iron acquisition systems. Protein names are reported in the legend.

Concerning microbial interactors, they were included in the evolutionary analysis if the following criteria were met: (i) the microbial molecule physically interacts with a mammalian heme-binding protein; (ii) a suitable number of sequenced genes (from different strains or species) encoding the microbial protein are available in public databases; (iii) the details of the interaction between the microbial molecule and the mammalian heme-binding protein(s) are known at the molecular level. These criteria restricted our analysis to the following microbial proteins: HpHbR from *Tripanosoma brucei*; IsdB and IsdH from *S. aureus/S. argenteus*; HpuA and HpuB from *N. meningitidis* and *N. gonorrhoeae* species; HasA from *P. aeruginosa;* and HxuA from *H. influenzae* (Figure 1).

### Evolutionary analyses in mammalian phylogenies

Coding sequences of *HBB* (Hb β subunit) and *HPX* (hemopexin) were retrieved from the Ensembl database and from the Nucleotide and Genome databases of National Center for Biotechnology Information (NCBI). A complete list of species analyzed for each gene and sequence accession IDs are reported in Supplementary Table S1.

cDNA alignments were performed using the RevTrans 2.0 utility (19). Manual editing was only used to correct a few misalignments in proximity of small gaps. Substitution saturation was checked using Xia's index implemented in DAMBE (20). This test compares a entropy-based index of saturation (I_ss_) with a critical value (I_ss.c_). If I_ss_ is significantly lower than I_ss.c_, sequences have not experienced substitution saturation. To further assess saturation across the gene phylogenies, we used the PAML (Phylogenetic Analysis using Maximum Likelihood) Free Ratio (FR) model to estimate dS for all branches (21).

Both alignments were screened for the presence of recombination breakpoints using GARD (Genetic Algorithm Recombination Detection) (22), a program that uses phylogenetic incongruence among segments of a sequence alignment to detect the best-fit number and location of recombination breakpoints.

The average non-synonymous substitution/synonymous substitution rate (dN/dS, also referred to as ω) was estimated using SLAC (Single-Likelihood Ancestor Counting) (23), a tool from the Hyphy package (24) based on a codon substitution matrix and ancestral state reconstruction.

To detect positive selection, we used the site models implemented in PAML package (25, 26). Specifically, we fitted site models that allow (M2a, M8) or disallow (M1a, M7, M8a) a class of sites to evolve with ω >1 to the data using the F3x4 and the F61 codon frequency models. Input trees were generated by maximum-likelihood using the program PhyML (27). Results were confirmed using the species tree as input (not shown).

Positively selected sites were identified using the Bayes Empirical Bayes analysis (BEB, from model M8 with a cutoff of 0.90) (28), the Fixed effects likelihood (FEL, with a default cutoff of 0.1) (23), and the Fast Unconstrained Bayesian AppRoximation (FUBAR, with a default cutoff of 0.90) (29). To limit false positives, we considered a site as positively selected if it was detected by at least two different methods.

We used the adaptive Branch-Site Random Effects Likelihood method (aBS-REL) to identify specific branches with a proportion of sites evolving with ω > 1. This method applies sequential likelihood ratio tests to identify branches under positive selection without *a priori* knowledge about which lineages are of interest (30); branches identified using this approach were cross-validated using the branch-site likelihood ratio tests from PAML (models MA and MA1). To identified sites evolving under positive selection on specific lineages we used the BEB analysis from MA (with a cutoff of 0.90) and the Mixed Effects Model of Evolution (MEME) (with the default cutoff of 0.1) (31). MEME allows the distribution of ω to vary from site to site and from branch to branch at a site. To limit false positives, only sites confirmed by both methods were considered as positively selected.

GARD (22), FEL (23), FUBAR (29), and MEME (31) analyses were performed either through the DataMonkey server (32) (http://www.datamonkey.org) or run locally (through HyPhy).

### Evolutionary analysis of pathogen-encoded interactors

Coding sequences for *HpHbR* from *Tripanosoma brucei, IsdB* and *IsdH* from *S. aureus*/*S. argenteus, HpuA* and *HpuB* from *N. meningitidis* and *N. gonorrhoeae* species, *HasA* from *P. aeruginosa*, and *HxuA* from *H. influenzae* were retrieved from NCBI Genome database. Detailed lists of strains analyzed for each genus is reported in Supplementary Tables S2–S7.

cDNA alignments were performed using the RevTrans 2.0 utility (19). Because *HpuA* is subjected to phase variation due to a stretch of polyG nucleotides at the beginning of the ORF, we aligned the cDNAs downstream this sequence (33).

Positive selection in *HpHbR, IsdB*, and *IsdH* was detected by application of the site models implemented in PAML (25, 26), as described above. BEB (28), FEL (23), and FUBAR (29) methods were applied to detect positively selected sites. This choice was motivated by the fact that different species were analyzed for these genes.

For *HpuA, HpuB, HxuA*, and *HasA* simultaneous inference of selection and recombination for analysis of positive selection was performed using omegaMap, a program based on a model of population genetics and molecular evolution (34). The program applies reversible-jump Markov Chain Monte Carlo (MCMC) to perform Bayesian inferences of ω and ρ (recombination parameter), allowing both parameters to vary along the sequence. An average block length of 10 and 30 codons was used to estimate ω and ρ, respectively. The set of priors is reported in Supplementary Table S8. For each alignment, two independent omegaMap runs, each with 1,000,000 iterations and a 50,000 iteration burn-in, were compared to assess convergence and merged to obtain the posterior probabilities.

### 3D structure analysis, homology modeling, and protein-protein docking

Protein 3D structures for HpHbR-Hb, IsdB-Hb, IsdH-Hb, HpuA-Hb, and Hxua-Hx were derived from the Protein Data Bank (PDB IDs: 5hu6, 5vmm, 4ij2, 5ee4, and 4rt6, respectively). The structures of HpuA of *N. meningitidis* and of N-terminal domain of human hemopexin were obtained by homology modeling using the *Kd*HpuA (PDB ID: 5ee4_A) and the rabbit hemopexin (PDB ID: 4rt6_B) structures as a template, respectively; analysis was performed through the SWISS-MODEL server (35). The accuracy of the models was examined through the GMQE (Global Model Quality Estimation) and QMEAN (Qualitative Model Energy ANalysis) scores (36). Because for HpuB no close homologs were found in PDB, the 3D model was derived using RaptorX server (37). The quality of the model was assessed by considering *p* value and uGDT (GDT) (unnormalized Global Distance Test).

For each complex, 3D-models were superimposed to homologous complex using PyMOL (The PyMOL Molecular Graphics System, Version 1.5.0.2 Schrödinger, LLC). The refinement of the rigid body orientations of the two binding partners and the optimization of side chain conformation were performed using the *docking_local_refine* docking protocol from ROSIE server (38, 39).

## Results and discussion

In mammals, the majority of body iron is contained within the protoporphyrin ring of the heme cofactor (40, 41). We thus focused on iron acquisition systems based on heme-piracy. In particular, we analyzed couples of host-pathogen interactors whose complexed 3D structures were solved by crystallographic techniques, or for which the molecular determinants of the interaction are known. The couples of host hemoproteins and pathogen-encoded heme acquisition systems analyzed herein are summarized in Figure 1.

### Adaptive evolution of hemoprotein genes in mammals

We first investigated the evolutionary history of genes encoding high-affinity heme-binding proteins in a large mammalian phylogeny. In humans, as well as in other vertebrates, Hb macromolecules in erythrocytes store more than two thirds of the body's iron content (40). In order to prevent oxidative damage following erythrocyte lysis, haptoglobin (Hp) and Hpx patrol the bloodstream for the presence of free Hb or free heme, respectively (42).

Hb has a tetrameric structure composed by two α and two β subunits. In Vertebrata, the α and β globin gene clusters originated by whole genome duplication and subsequent gene tandem duplication events from a common ancestral globin gene (43). Herein, we wished to gain further insight into the evolution of Hb in mammalian phylogeny by inter-specific comparison of orthologous genes. Although the synteny across the two globin gene clusters is generally conserved in mammals, we excluded from our analysis the α subunit genes (*HBA1* and *HBA2*) because 2 or 3 functional copies exist in the majority of mammalian species, making it difficult to assign correct orthology and paralogy relationships among duplicates (16). Similarly, the *HP* gene was not included in the study due to the extensive copy number variation in humans (17, 18).

Taking all these issues into account, we decided to focus our analyses on the *HBB* (Hb β subunit) and *HPX* (hemopexin) genes. We retrieved coding sequence information of placental mammals belonging to the Euarchontoglires, Laurasiatheria, and Afrotheria superorders. For *HBB* we excluded species from Eulipotyphlans, Carnivores, Cetaceans, and some Microchiropteran bats, as these lineages present a chimeric *HBB/HBD* fusion gene primarily responsible for hemoglobin β type subunit synthesis (44) (Supplementary Table S1). Because recombination can generate false positive results (45, 46), sequence alignments were screened for recombination using GARD (22). No recombination breakpoint was detected for *HBB* or *HPX*.

No evidence of significant saturation was obtained for any alignment (Supplementary Table S9). Furthermore, no branches showed dS ≥ 1 in either alignment.

We calculated the average non-synonymous substitution/synonymous substitution rate ratio (ω) using SLAC (23). As expected, ω values were lower than 1, indicating purifying selection as the major driving force in shaping *HBB* and *HPX* gene diversity (12) (Supplementary Table S10).

We thus applied the likelihood ratio tests (LRT) implemented in the *codeml* program (26) to test whether positive selection acted on a restricted subset of codons. For both genes, neutral models were rejected in favor of positive selection models, indicating that some codons evolved with ω > 1 (positive selection). These results were confirmed under two different codon frequency models (Supplementary Table S11). Positively selected sites in *HBB* and *HPX* were identified by applying three different methods: BEB, FUBAR, and FEL. A conservative approach was adopted and only sites identified by at least two methods were considered to be positively selected (Supplementary Table S11).

**Figure 2:**
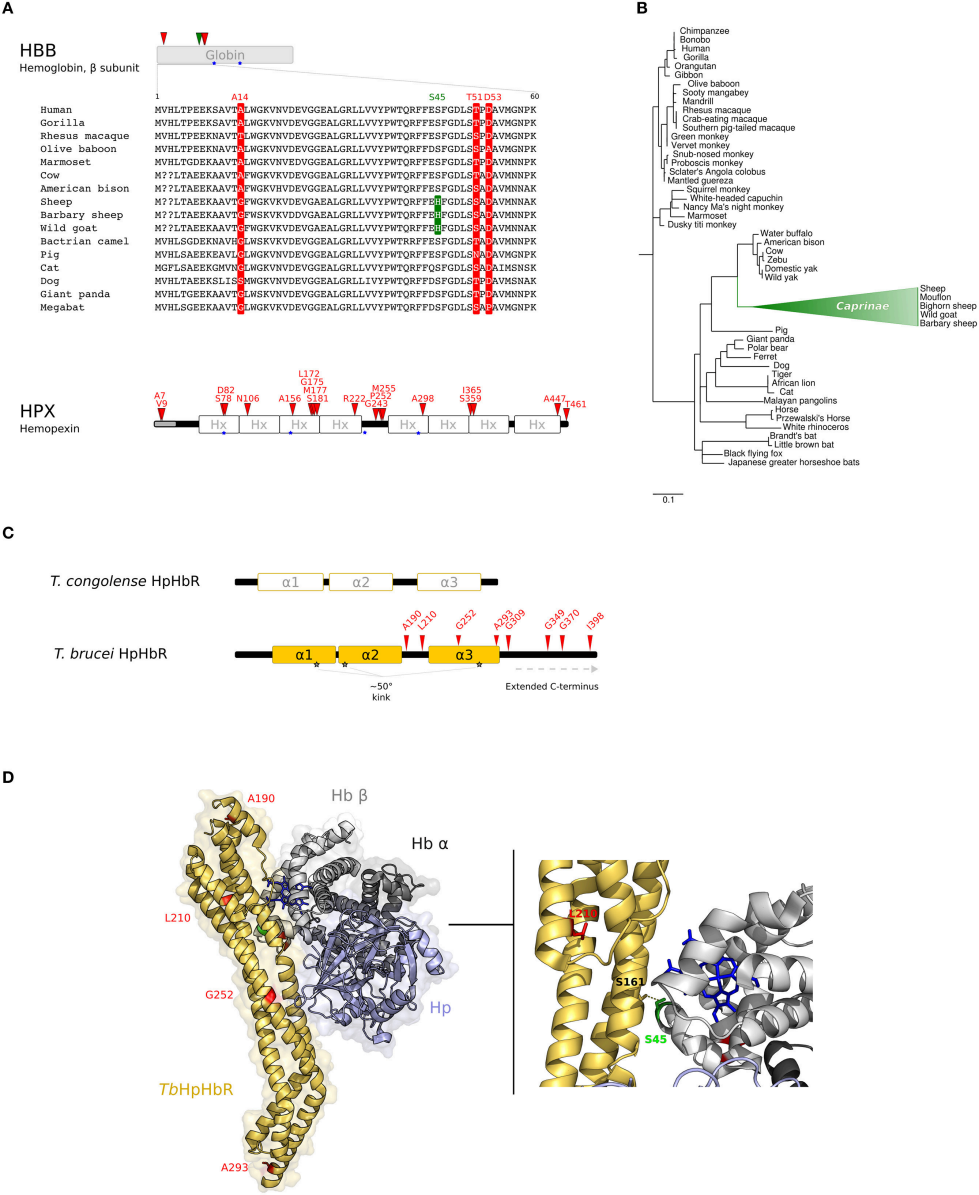
Evolution at the interaction surface between Hb and *Trypanosome brucei* HpHbR. **(A)** Positively selected sites are mapped onto the β chain of Hb (*HBB* gene product) and on Hpx. A multiple alignment of the β chain of Hb (amino acids 1–60) for a few of representative mammalian species is shown. Sites that are positively selected in the mammalian phylogeny are marked in red; the site selected on the Caprinae branch is in green. Heme binding sites are indicated as blue stars. **(B)** aBS-REL analysis of positive selection for *HBB* in mammals. Branch lengths are scaled to the expected number of substitutions per nucleotide. The Caprinae branch is in green. **(C)** Schematic representation of trypanosome HpHbRs. Positively selected sites are shown in red. **(D)** 3D structure of human HpHb bound to *Tb*HpHbR (PDB code: 5hu6); Hb is shown in gray (light, β subunit; dark, α subunit), Hp in light-blue, and HpHbR in light orange. Positively selected sites are mapped onto the structure; those that located at the contact interface are indicated in the enlargement. Hb-bound heme molecules are represented as blue sticks.

For *HBB*, three positively selected sites were identified (Figure 2A, Supplementary Table S11). All these sites are surface-exposed and are not involved in the interaction with α subunits, nor in the coordination of heme. For *HPX*, 19 positively selected sites were found, spanning throughout the protein sequence (Figure 2A, Supplementary Table S11).

The branch site-random effects likelihood (aBS-REL) method (30) was next adopted to analyse possible variations in selective pressure along specific branches. The Caprinae branch showed statistically-supported evidence of positive selection in the mammalian phylogeny for *HBB* (Figure 2B). This result was cross-validated and confirmed using the *codeml* branch-site LRT models (47) (Supplementary Table S12). Position 45 was identified as positively selected along the Caprinae branch (Figure 2A). This site was determined through BEB analysis (47) and with MEME, a method specifically developed to detect episodic positive selection (Figure 2A, Supplementary Table S12).

For *HPX*, episodic positive selection was detected in the great roundleaf bat and in the dolphin lineages, but no positively selected site was detected for either species (Supplementary Table S12).

These data indicate that although purifying selection represented the major evolutionary force, Hb and Hpx, two highly abundant housekeeping proteins, were positively selected during the evolution of placental mammals. In this respect, it is worth noting that proteins involved in central homeostatic processes are expected to be strongly constrained to preserve their function, suggesting that a minority of sites will be able to evolve in response to pathogen-driven selection without causing an important loss of fitness (12). Emblematic in this respect is the sickle cell mutation in *HBB* (HbS allele), which confers extremely strong protection against severe malaria to heterozygotes (48), but causes homozygotes to suffer severe symptoms and premature death (48). How the HbS and other structural Hb variants (i.e., HbC and HbE) protect from malaria is still unclear but the mechanisms seem to be unrelated to nutritional immunity, despite the use of Hb as a source of amino acids by *Plasmodium* parasites (48, 49). Clearly, it is possible that some of the selected sites we identified in *HBB* evolved in response to the selective pressure exerted by mammalian *Plasmodium* parasites. However, other pathogens exerted important selective pressure on human populations and, more generally on their mammalian hosts (see below). To assess whether the competition with heme acquisition systems also played a role in the evolution of Hb and Hpx, we analyzed the evolutionary history of microbial-encoded interactors of these two hemoproteins. We relied on structural modeling to infer the selective events at the binding interfaces.

### The haptoglobin-hemoglobin (HpHb) receptor from african trypanosomes

Trypanosomes are eukaryotic unicellular parasites with a complex life-cycle, switching between mammalian and insect hosts. Among African trypanosomes, *T. brucei brucei, T. congolense* and *T. vivax* infect both domesticated and wild mammals but are unable to infect humans and most other primates because they are susceptible to two primate-specific Trypanosome Lytic Factors (TLF1 and TLF2). In humans, African sleeping sickness is caused by two *T. brucei* subspecies, *T. b. gambiense* and *T. b. rhodesiense*, which evolved different strategies to escape human TLFs. *T. b. rhodesiense* expresses human serum resistance associated (SRA) protein (50), whereas group 1 *T. b. gambiense* escapes human immune response by a multifactorial mechanism that includes the reduction of TLF1 uptake (51–53), which is mediated by the haptoglobin-hemoglobin (HpHb) receptor (*Tb*HpHbR) (54). In fact, *Tb*HpHbR is expressed at low levels in group 1 *T. b. gambiense* and the receptor has acquired a mutation (L210S) that strongly reduces its affinity for TLF1 (55–59). Therefore, in this parasite, heme piracy and immune evasion are intertwined processes.

Recent evidence suggested that *Tb*HpHbR evolved from an ancestral Hb-binding receptor expressed in the epimastigote stage of *T. congolense* and *T. vivax* (60). In parallel with its change in expression pattern, *Tb*HpHbR acquired higher affinity for HpHb than for free Hb (which is not present in the blood stream) and gained an extended C-terminal domain and a ~50° kink in the three α-helical bundle structure. Both elements are suggested to favor the exposure of the receptor onto the cell-surface, that is coated by a dense layer of variant surface glycoproteins (VSG) to avoid immune clearance (60, 61) (Figure 2C).

To investigate whether positive selection contributed to the evolution of HpHbR, we aligned the coding sequence of the receptor from 67 trypanosome strains, including *T. b. rhodesiense, T. b. gambiense, T. b. brucei, T. evansi*, and *T. equiperdum* (Supplementary Table S2). *HpHbR* sequences from other Trypanosome subgenera, i.e., *T. congolense* and *T. vivax*, were excluded due to excessive divergence resulting in unreliable alignments. We note that the high sequence identity of the *T. evansi* and *T. equiperdum* genes to the *T. brucei HpHbR* sequence (>98.5% identity) indicates that they represent orthologs. However, the binding specificity for HpHb of these receptors has never been investigated.

No evidence of recombination was detected with GARD. Positive selection was tested as described above and significant evidence was obtained, with 8 positively selected sites detected by at least two methods (Figure 2C, Supplementary Table S13).

Notably, we identified L210 as a target of positive selection. This residue is packed in the hydrophobic core of the receptor head (Figure 2D) and the substitution also leads to a reduced affinity for HpHb, suggesting an overall destabilization of the head region, influencing the conformation of the ligand binding site (58, 62). Thus, the selective advantage conferred by TLF1 resistance is traded off by *T. b. gambiense* with decreased iron uptake. However, mutagenesis experiments indicated that the L210S substitution totally abolishes TLF1 binding even at high concentrations, whereas affinity for HpHb is decreased but binding still occurs (58). Hence, the L210S selected site shifts the balance between immune escape and nutritional needs to favor the parasite.

As for the other positively selected sites we detected in HpHbR, three of these are located in the extended C-terminal region of TbHpHbR (Figure 2C).

Analysis of the three-dimensional structure of *Tb*HpHbR in complex with HpHb (59) also indicated that residue S45 in the Hb β subunit, which is positively selected in the Caprinae branch, lies at the receptor-binding interface and was specifically reported to stabilize the complex by a hydrogen bond with S161 of *Tb*HpHbR (59, 62) (Figure 2D).

Interestingly, species belonging to Caprinae family are susceptible to trypanosome infection (63). In particular, African goats can be infected by a wide range of trypanosome species, but the course of the disease is often mild or even sub-clinical (63). Because parasitemia is usually low but persistent, African goats are thought to have developed mechanisms of trypanotolerance. African trypanosomes are heme auxotrophs; as the S45H substitution in Caprinae species is likely to decrease HpHb binding by trypanosomes, it may in turn influence the persistence/severity of *T. brucei* infection. Experimental studies to validate this hypothesis would be worthwhile, as small-ruminants are likely to represent an important reservoir of trypanosomes and caprine trypanosomosis is considered an important factor in programs of disease prevention and control (63).

Concerning humans, African trypanosomes are considered to have acted as an important selective pressure. This is testified by the observation that selection drove the frequency increase of coding variants in the human *APOL1* gene (which encodes a component of TLFs) in Africans (64). These variants confer high lytic activity against *Trypanosoma brucei rhodesiense*, but predispose to kidney disease (64). The fact that, with the exclusion of the Caprinae branch, we did not detect Hb selected sites at the interaction surface with *Tb*HpHbR is not in contrast with these observations. As mentioned above, structural/functional constraint limit the possibilities of Hb adaptive evolution. Also, in analogy to the APOL1 variants, sites that evolved in response to *T. brucei-*mediated selective pressure may have not reached fixation in human populations and would therefore go undetected in the analyses we performed. Finally, selected sites at the *Tb*HpHbR interface may be located in Hp, which we could not analyse.

### Heme acquisition systems in gram-positive bacterial pathogens

Several Gram-positive bacteria use the Iron-regulated Surface Determinant system (Isd) to recruit host hemoproteins, to extract heme molecules, and to funnel them to a permease across the cell membrane (7, 65, 66). *Isd* genes encode proteins anchored to the cell-surface and containing NEAT (near transporter) domains responsible for heme and/or hemoprotein binding. The Isd-dependent heme uptake system was well characterized in the *Staphylococcus* genus. This system is composed of 9 different Isd proteins, named “A” through “I”; IsdB and IsdH are the primary receptors for Hb and HpHb complexes, respectively (67) (Figure 1).

We retrieved and aligned *IsdB* and *IsdH* coding sequences of strains belonging to the *S. aureus* and to *S. argenteus* species, which represent a major cause of human clinical disease (68) (Supplementary Table S3). Due to the high phenotypic similarity, *S. argenteus* has often been misclassified as *S. aureus*, and it was only recently identified as a new species (68, 69).

**Figure 3:**
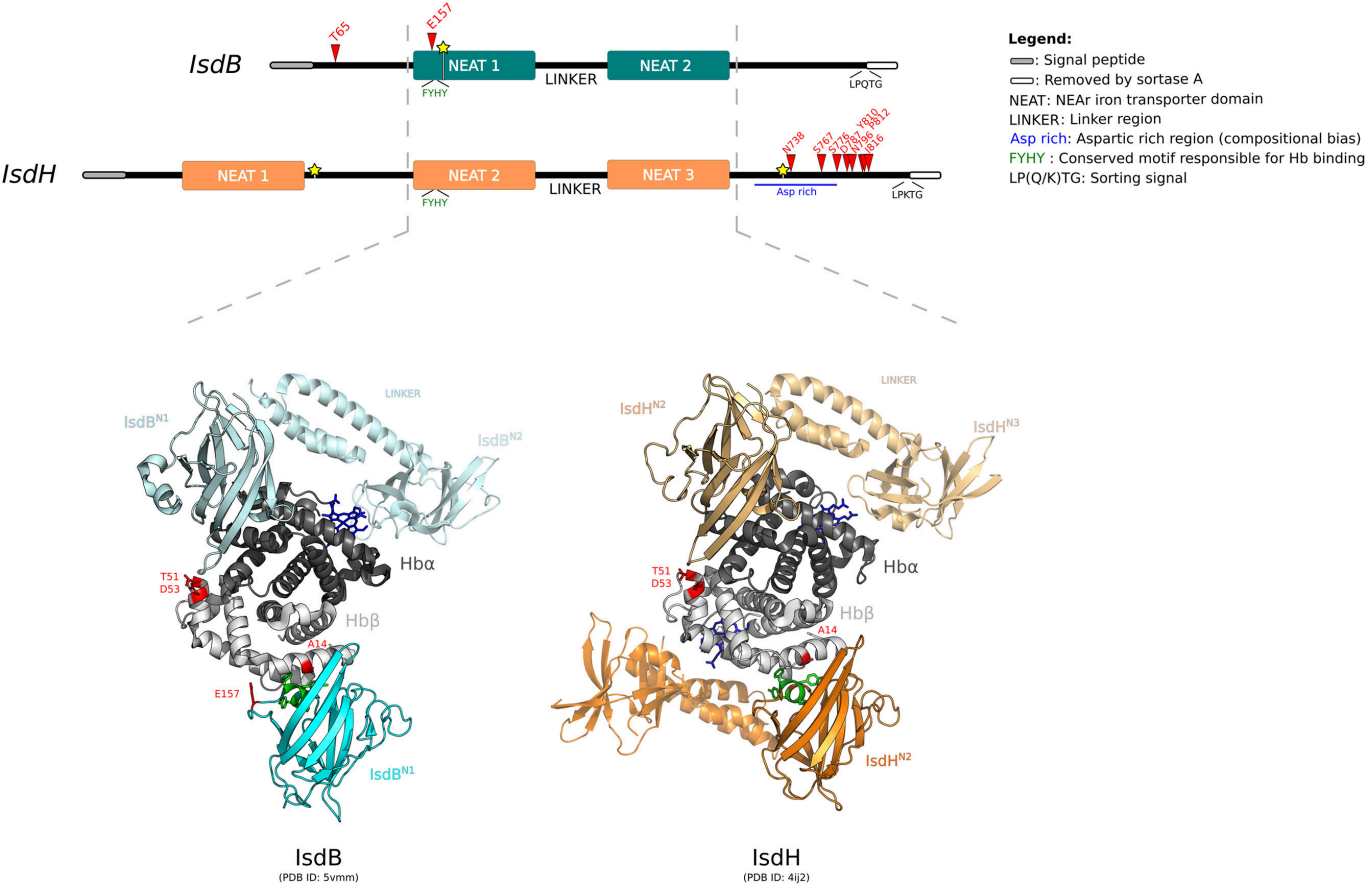
Positive selection at the interaction surface between Hb and *S. aureus* IsdB/H. A schematic representation of the domain structure of IsdB and IsdH is shown. Domains are color-coded as reported in the legend (left). The highly homologous regions between the two receptors are indicated by the hatched lines. Positively selected sites are reported in red, recombination breakpoints as yellow stars. The 3D structures of human Hb in complex with IsdB^N1−linker−N2^ (cyan) and IsdH^N2−linker−N3^ (orange) are shown (PDB IDs: 5vmm and 4ij2). Hb is color-coded as in Figure 2. In both representations, two Isd proteins are reported, bound to the α and β Hb chains. The first NEAT domain is contoured, whereas the linker region and the second NEAT domain, if solved in the crystal, are in transparency. Positively selected sites are in red, the conserved -FYHY- motif responsible for Hb binding is in green.

GARD detected one breakpoint in *IsdB* and two breakpoints in the *IsdH* gene (Figure 3). Positive selection was tested independently for the sub-regions of both genes, split accordingly to the location of recombination breakpoints.

For *IsdB* region 1, the neutral models were rejected in favor of the positive selection models (after Bonferroni correction for two tests, to account for alignment splitting) (Supplementary Table S13). In this region, two sites were identified as positively selected, T65 and E157 (Figure 3). This latter site is located on loop 2 of the NEAT-1 domain (IsdB^N1^), which displays high homology to IsdH^N2^, for which the 3D structure in complex with hemoglobin was solved (70, 71). Based on this structure, E157 is located at the binding interface with Hb (Figure 3). Indeed, this residue lies just upstream the aromatic residues -FYHY- (Figure 3), in a region presenting a high degree of flexibility. Notably, this region was shown to modulate the strength of hemoglobin binding and of heme capture, as observed by comparing the affinity for Hb in IsdB mutagenesis studies (71, 72).

T65 is in the N-terminal segment of IsdB, for which structural information are unavailable (Figure 3). Although this domain was not reported to be directly involved in hemoglobin interaction, together with NEAT-1 it enhances the heme-transfer from oxidized hemoglobin (metHb) to NEAT-2 domain, affecting the enzymatic kinetic of heme assimilation (73).

For *IsdH*, evidence of positive selection was detected only in the terminal region, with 8 sites identified as positively selected (Figure 3, Supplementary Table S13). All these sites are located downstream the third NEAT domain, spanning throughout an aspartic acid-rich region before the sortase cleavage site. Unfortunately, no structural or functional data have been reported for this protein region (Figure 3) (70, 74). This C-terminal (Ct) fragment is present also in other Isd components anchored to the cell wall. Among Isd proteins, the Ct portion has variable length and may act as a spacer to position Isd proteins sequentially onto the cell wall, thus enabling the correct heme recruitment and its relay across the membrane (73).

IsdB and IsdH are homologous and bind hemoproteins with a similar mechanism, despite the differences of substrate specificity for Hb or HpHb complexes, respectively. Both crystallographic and kinetic data have demonstrated that Hb capture by the IsdB/H NEAT domains occurs with a similar mechanism for the α and β chains, although Hb β chain binding is weaker (70, 73, 75). Notably, we observed that A14, detected as positively selected in the mammalian phylogeny for *HBB*, lies at the interaction surface with IsdB^N1^, as well as with IsdH^N2^ (Figure 3) (70, 75). Positively selected sites T51 and D53 contribute to form an exposed region on the Hb β chain and face IsdB^N1^/IsdH^N2^ when the α chain is bound (Figure 3). However, analysis of atomic distances suggests that these residues are not directly involved in complex formation.

Hb binding by IsdB (but not by IsdH) is strictly required for *S. aureus* hemoglobin-derived iron acquisition and virulence (72, 76). IsdB specifically recruits heme from oxidized hemoglobin (metHb), which is released in the bloodstream when bacterial-secreted toxins cause erythrocyte lysis (77). The high-affinity of IsdB for human metHb allows its utilization as preferred iron source during the early phase of staphylococcus infection, leading to host colonization (78).

*S. aureus* is a human-specific pathogen and approximately 25–30% of healthy humans are persistently or intermittently colonized with *S. aureus* (79). This figure was most likely higher in the past (80) and nasal carriage represents a risk to develop staphylococcus-associated diseases (81). IsdB binds human Hb with increased efficiency compared to Hb from other mammalian species, suggesting a specific adaptation to the human host (82). Thus, interspecies variation at site A14 may affect hemoglobin capture by staphylococcal IsdB and contribute to determine its host range and/or pathogenicity. Indeed, mice expressing human hemoglobin are more susceptible to systemic infection from *S. aureus* strains that carry an intact *IsdB* gene, but not from Δ*IsdB* strains (82).

Interestingly, experiments in mice have shown that anti-IsdB (and anti-IsdA) antibodies which interfere with heme binding protect the animals against abscess formation and lethal challenge. This effect is not mediated by increased clearance of the pathogen via opsonophagocytic killing (83). Conversely, protection seems to be mediated by the abolition of the physiological functions of IsdA/IsdB, namely heme scavenging from hemoglobin (83). This observation provides insight on an aspect of host-pathogen interactions that is extremely relevant from a medical perspective, namely the targeting of pathogen virulence, as opposed to approaches that rely on microbial killing (84). Indeed, most microbial iron acquisition systems are not necessary to establish host colonization but represent virulence factors (14) (see also sections below). The control of pathogen virulence or the elicitation of host tolerance (e.g., damage limitation mechanisms) are regarded with increasing interest as possible therapeutic interventions, as they are not expected to select for pathogen populations resistant to drugs or vaccines (84). Heme scavengers and siderophores may represent attractive candidates for such approaches.

### Heme acquisition systems in gram-negative bacterial pathogens

The outer membrane of Gram-negative bacteria represents an additional barrier to heme acquisition. Gram-negatives have thus evolved elaborate heme-uptake systems, including outer-membrane receptors for host hemoproteins and secreted hemophores (7).

#### Neisseriaceae

Bacteria from the *Neisseriaceae* family present different receptor systems for heme uptake: HmbR and/or HpuAB. HmbR specifically extracts heme from hemoglobin, whereas HpuAB can extract heme from Hb-Hp complexes, as well (85, 86) (Figure 1). The molecular evolution of *HmbR* in *Neisseria meningitidis* was already investigated, demonstrating that positive selection mainly targeted portions of the receptor predicted to be surface-exposed (87). To date, very little is known about HmbR structure and the mechanisms of hemoglobin recruitment and heme transport and only some residues likely involved in heme coordination have been identified (88).

We thus focused on the HpuAB system, which is composed of two proteins, HpuA and HpuB, whose expression is finely controlled by an iron-repressed operon (89). HpuA, a lipoprotein anchored to the outer membrane, is required for the high-affinity interaction between Hb and HpuB, which is the TonB-dependent receptor (90). The high-resolution 3D-structure of HpuA from *Kingella denitrificans (Kd*HpuA*)* in complex with human Hb was recently solved, indicating a direct interaction between the two proteins (91).

Although no structural or functional data are available for HpuB, we extended our evolutionary analyses to this gene for the sake of completeness.

A previous work analyzed the genetic diversity of this system in a relatively small phylogeny including both pathogenic and non-pathogenic *Neisseria* species. The authors interpreted patterns of HpuA/B diversity in terms of immune selection (92).

Because recombination is high in *Neisseriaceae* (93), the *HpuA* and *HpuB* coding sequences from *N. meningitidis* and *N. gonorrhoeae* species (Supplementary Tables S4,S5) were analyzed separately with omegaMap, a population genetics method that simultaneously estimates recombination rate and selection. In particular, selection is inferred in terms of ω estimation, better described in this case as the relative occurrence of non-synonymous and synonymous polymorphisms (34). The HpuAB system is subject to mononucleotide repeat-mediated phase variation (33). We analyzed the region downstream the repeat tract in HpuA, irrespective of the sequence phase.

**Figure 4:**
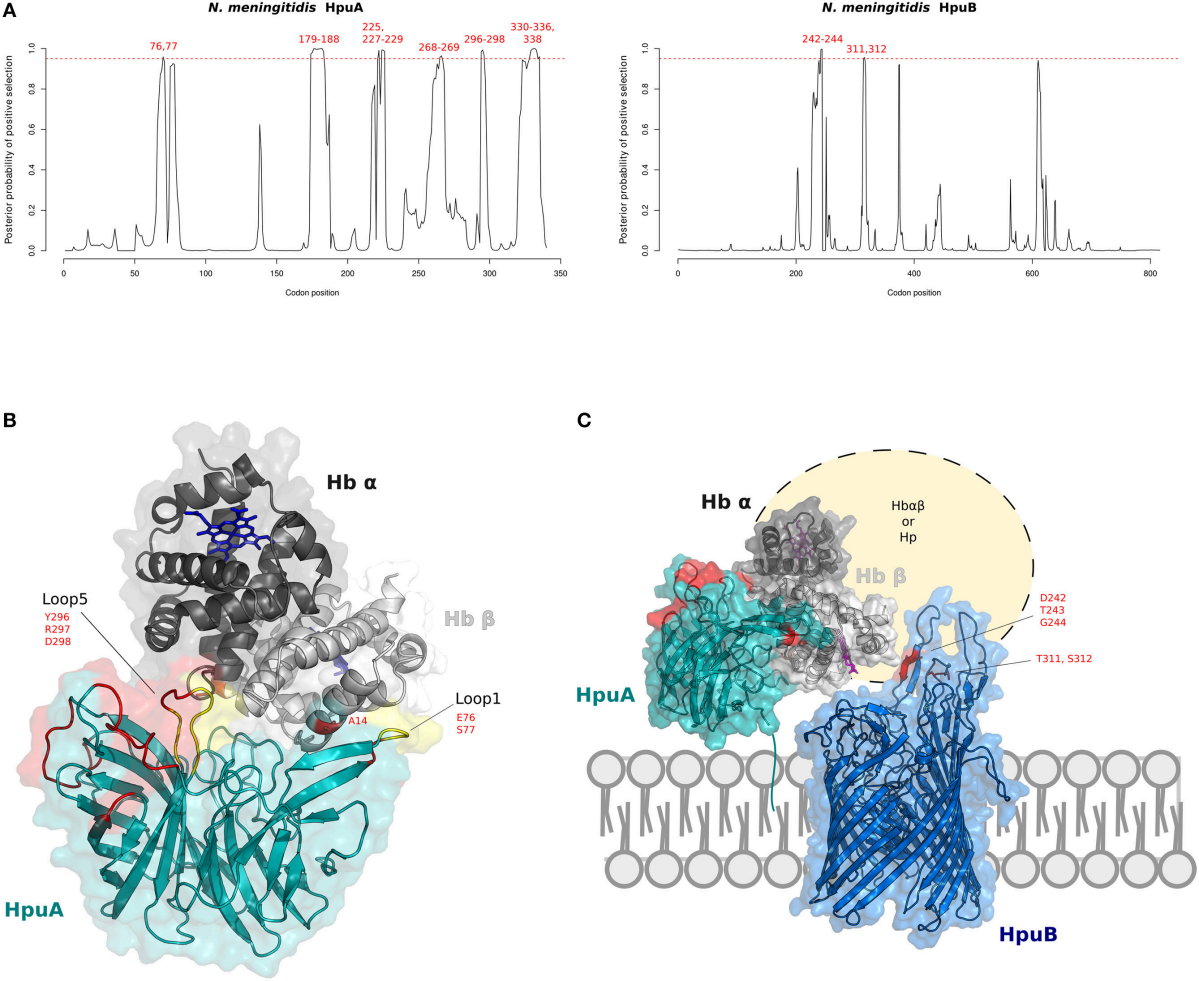
Positive selection at the interaction surface between Hb and *N. meningitidis* HpuA/HpuB. **(A)** omegaMap results: the posterior probability of positive selection (ω>1) along *N. meningitidis* HpuA and HpuB coding sequences are plotted. The hatched red lines correspond to a posterior probability of selection equal to 0.95. Positively selected codons are in red. Codon positions refer to *N. meningitidis* FAM18 strain (HpuA ID:A1KW50; HpuB ID: A1KW51). **(B)** The 3D structure of human Hb in complex with *N. meningitidis* HpuA (model, cyan). Hb is color-coded as in Figure 2. HpuA loops 1 and 5, involved in Hb interaction, are in yellow. Positively selected sites are in red. Positively selected sites located at the contact interface are labeled. Hb-bound heme molecules are represented as blue sticks. **(C)** Model of proposed cooperation between HpuA (cyan) and HpuB (model, blue) for heme import, associated with the membrane. Positively selected sites are in red, Hb-bound heme molecules are represented as violet sticks.

No signal of positive selection was observed in *N. gonorrhoeae* species for either gene. Conversely, many positively selected sites were detected in *N. meningitidis*, both for *HpuA* and for *HpuB* (Figure 4A). Our results for *N. meninigitidis* are in agreement with those reported by Harrison and colleagues (92) for Family B *Neisseriaceae*, strongly suggesting that the signals of selection they detected were accounted for by *N. meningitidis* isolates.

We thus reconstructed the structural model of HpuA of *N. meningitidis* FAM18 strain by homology modeling. The *Kd*HpuA (chain A), complexed to Hb, was used as template (91) (Supplementary Table S14). A local docking refinement was performed to find the optimal fit between the *Nm*HpuA model and human Hb. Positively selected sites were mapped onto the best-scored structure (ranked by interaction energy). All positively selected sites are located on the long surface-exposed loops (Figure 4B). These sites include residues in loop1 and loop5, which are involved in Hb interaction. In particular, loop1 primarily influences affinity for Hb in *Kd*HpuA (91).

As with HpuA, we reconstructed the three-dimensional structure of *N. meningitidis* HpuB (FAM18 strain) (Figure 4C). Because sequences in PDB database display very low identity with *Nm*HpuB, the model was reconstruced with the RaptorX server, reaching a good quality assessment. The server identified the crystal structure of transferrin binding protein A (TbpA) of *N. meningitidis* in complex with C-terminal domain of human transferrin (Supplementary Table S15) as best template. *Nm*TbpA is an integral outer membrane protein belonging to the family of Ton-B depend transporters. Positively selected sites of HpuB in *N. meningitidis* strains were mapped onto the structure, confirming that positive selection acted on surface exposed loops likely involved in Hb and/or Hpua recognition (Figure 4C). Indeed, similar results were obtained by Harrison and coworkers with a different HpuB model reconstructed by homology modeling using ShuA from *Shigella dysenteriae* as the template (92).

In *N. meningitidis*, the HpuAB and the HbmR systems are thought to be involved in pathogenicity, as most disease-associated meningococcal strains encode one or both Hb receptor systems, and clonal complexes causing high disease rates encode both HpuAB and HmbR (33). Results herein indicate that HpuA and HpuB, evolved under strong positive selection in *N. meningitidis* and that several HpuA selected sites are located in loops that determine Hb binding. As mentioned above, previous reports on the evolution of HpuA/B in Neisseriaceae suggested that the host immune system shaped diversity at the surface-exposed loops. We do not exclude that immune selection contributed to the evolution of Hb receptors. As in the case of *Tb*HpHbR, the same sites might modulate both immune evasion and iron acquisition. Indeed, this is most likely also the case for TbpA, which was previously shown to be engaged in a genetic conflict with primate transferrin (13). Most positively selected sites in the TbpA proteins from *H. influenzae* and *N. gonorrhoeae* are located on exposed loops, but only some of them are within the binding interface with transferrin (13), suggesting that immune selection also contributed to shape TbpA diversity.

In contrast with the strong selection observed in meningococcal strains, we did not detect positive selection at HpuA/B in *N. gonorrhoeae*. The two *Neisseria* species colonize distinct ecological niches in the human host and display remarkably different dissemination routes. Thus, the selective pressure acting on iron acquisition systems may vary depending on the relative abundance of available sources. In fact, most gonococcal strains isolated from human patients are phase off for HpuA/B with the exclusion of those deriving from women in their early menses (94). Moreover, *N. gonorrhoeae* strains isolated to date do not express HmbR, which is present as a frame-shifted pseudogene (95). Overall, these observations suggest that gonococci are less dependent on Hb as an iron source than meningococci and thus that the selective pressure acting on HpuA/B is weak in *N. gonorrhoeae*.

#### Haemophilus influenzae

Among Gram-negative bacteria, *Haemophilus influenzae* is a heme auxotroph human commensal/pathogen. To sustain its aerobic growth it developed different strategies to acquire heme from different host sources. In addition to Hb, *H. influenzae* also targets hemopexin (Hpx) as a host heme source. Hpx is present at relatively low concentrations in body fluids, but it has a very high affinity for heme (96). *H. influenzae* HxuA is a hemophore displaying high-affinity binding for Hpx (97, 98) (Figure 1). Although *H. influenzae* is uniquely found in human hosts, HxuA can accept heme from both human and rabbit hemopexins (99).

**Figure 5:**
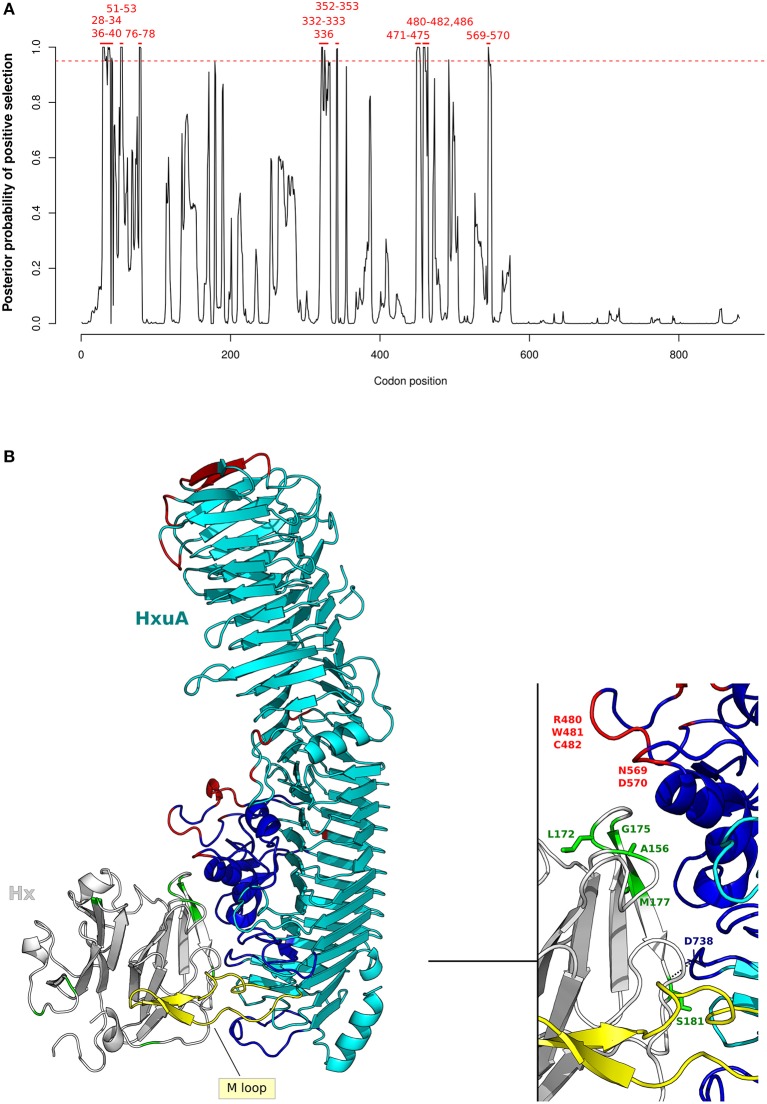
Positive selection at *H. influenzae* Hxua-Hpx interaction surface. **(A)** omegaMap results for HxuA are shown as in Figure 4A. Codon positions refer to *H. influenzae* Rd KW20 strain (ID:P44602), used as reference. **(B)** Cartoon representation of HxuA (cyan) in complex with the N-terminal domain model of human Hpx (light gray). The HxuA secondary structure elements that provide residues involved in Hpx interaction are in blue, the M-loop in yellow, HxuA positively selected sites in red, and Hpx positively selected sites in green. Positively selected sites in Hpx that are located at the contact interface are indicated in the enlargement.

By applying omegaMap, we detected several signals of positive selection for *H. influenzae* HxuA (Figure 5A, Supplementary Table S6). The crystal structure of HxuA in complex with the N-terminal domain of rabbit hemopexin was recently solved (100). Starting from this structure, we used homology modeling to reconstruct the three-dimensional model of human hemopexin N-terminal domain (Supplementary Table S14). The model was then superimposed onto the rabbit Hpx structure and an optimization of the lateral chains of the two binding partners was performed.

Results indicated that HxuA positively selected sites (100) are mainly located at the mobile junctions of the three parallel β-sheets that define the right-handed β-helix structure of HxuA. In particular, positive selection targeted the first two β-strands at the N-terminus, as well as two long insertions containing α-helix elements that are responsible for Hpx binding (100) (Figure 5B).

As for Hpx, five positively selected sites (A156, L172, G175, M177, and S181) are located at the interaction surface with HxuA. Specifically, in our human Hpx model, S181 stabilizes the complex through a polar bond with the carbonil group of D738 in HxuA, whereas A156 contributes to the binding with an hydrophobic interaction (Figure 5B) (100).

Overall, these results suggest that Hpx and HxuA evolved in the context of a genetic conflict, as evidence of positive selection was found for both interactors at the binding interface. No positively selected sites were found in the HxuA M-loop that is responsible for heme-scavenging from Hpx, suggesting that selection mainly acted to establish and maintain Hpx binding.

*Haemophilus influenzae* is an obligate commensal/pathogen and its host range is restricted to humans. The bacterium asymptomatically colonizes the nasopharynx and is absolutely dependent on host-derived heme for its aerobic growth. The HxuA/B/C gene cluster is a virulence factor for *H. influenzae* (101), which is one of the few bacterial species that can utilize Hpx as an iron source. HxuA may therefore allow *H. influenzae* to successfully compete with other microbial colonizers for iron acquisition.

Notably, *H. influenzae* and related bacteria are likely to have exerted a considerable selective pressure on humans and on other mammals. This bacterium represents the second most common cause of childhood pneumonia, a disease that accounted for 16% of all deaths of children under 5 years in 2015 (http://www.who.int/mediacentre/factsheets/fs331/en/, Updated September 2016). Moreover, *H. influenzae* belongs to the *Pasturellaceae* family, which includes several other pathogenic species for humans and animals. Among these, *Mannheimia haemolytica* (102) and *Haemophilus parasuis* (103) express HxuA homologs, suggesting that the arms race between HxuA and HPX is long-standing.

#### Pseudomonas aeruginosa

As *H. influenzae, P. aeruginosa* encodes a specific hemophore uptake system. Together with a direct system for heme uptake called Phu (*Pseudomonas* heme uptake), the bacterium secretes the HasA (Heme assimilation system) hemophore, which targets Hb and free heme molecules (Figure 1). This receptor has been characterized and the structural determinants for heme coordination and Hb binding were defined (104, 105). *HasA* coding sequences for 94 *P. aeruginosa* strains (Supplementary Table S7) were aligned and analyzed by omegaMap as described above. Unlike all other heme scavengers analyzed, no signals of positive selection were detected. The reasons for this are probably manifold. First, *HasA* targets both Hb and free heme, suggesting that the selective pressure for Hb recognition is relatively weak. Second, *P. aeruginosa* possesses a plethora of systems for iron acquisition, targeting not only hemoproteins and heme, but also ferrous iron (*feo* system) (106) and ferric cytrate (*fec* system) (107). Moreover, as it is the case of *S. aureus, P. aeruginosa* possesses siderophore-based systems (108) and a heme biosynthetic pathway (109). Heme synthesis is necessary to *P. aeruginosa* fitness, as observed in mutants inactivate for *HemY*, a heme biosynthesis gene, which are unable to colonize the murine gastrointestinal tract (110). Notably, within-host selection of mutations in the promoter of the Phu system occur and confer a growth advantage during chronic infections in cystic fibrosis patients (111). Thus, the redundancy of molecular strategies to cope with iron-limitation in *P. aeruginosa* (112), most likely results in no or mild selective pressure on HasA.

## Conclusions

It is becoming increasingly clear that host-pathogen genetic conflicts are not confined to genes directly involved in immune response, but extend to loci that, for different reasons, encode molecules interacting with viral or microbial components (12). For instance, signatures of pathogen-driven positive selection were described for housekeeping proteins that function as incidental viral receptors (12). Proteins involved in nutritional immunity represent another class of molecules that play essential roles unrelated to “classical” host defense, but also interact with pathogen-encoded proteins to avoid micro-nutrient piracy. Evidence that genes involved in nutritional immunity can be targets of positive selection was first obtained for transferrin and more recently for lactoferrin (13, 113). We now extend these observations by showing that Hb and Hpx represented positive selection targets during mammalian evolution and that the selective pressure was most likely exerted by pathogenic microrganisms, which in turn evolved to subvert nutritional immunity.

Indeed, we detected selected sites at the interaction surface with mammalian hemoproteins in several molecules encoded by pathogenic microorganisms, both prokaryotic and eukaryotic, and carrying extremely different heme acquisition systems. This suggests that the molecular arms race for iron piracy is widespread and additional players will most likely be described in the future.

## Author contributions

MS, AM, and RC conceived and designed the study. AM performed evolutionary analyses, homology modeling and docking studies, and produced the figures, with input from all authors. AM, DF, MC, and RC analyzed the data. RC and DF provided support during the bioinformatics analyses. MS and AM wrote the manuscript, with critical input from MC and from the remaining authors. All authors read and approved the final manuscript.

### Conflict of interest statement

The authors declare that the research was conducted in the absence of any commercial or financial relationships that could be construed as a potential conflict of interest.

## Abbreviations

Hb: hemoglobin
Hp: haptoglobin
Hpx: hemopexin
TLF: trypanosome lytic factor
HpHb: haptoglobin-hemoglobin complex
GARD: Genetic Algorithm Recombination Detection
SLAC: Single-Likelihood Ancestor Counting
ω (or dN/dS): non-synonymous substitution/synonymous substitution rate ratio
PAML: Phylogenetic Analysis using Maximum Likelihood
BEB: Bayes Empirical Bayes
FUBAR: Fast Unconstrained Bayesian AppRoximation
FEL: Fixed effects likelihood
aBS-REL: adaptive Branch-Site Random Effects Likelihood
MEME: Mixed Effects Model of Evolution
ORF: open reading frame
MCMC: Markov Chain Monte Carlo
GMQE: Global Model Quality Estimation
QMEAN: Qualitative Model Energy ANalysis
uGDT: unnormalized Global Distance Test
LRT: Likelihood Ratio Test
SRA: serum resistance associated
NEAT: NEAr Transporter domain
metHb: Methemoglobin
HbS: Sickle hemoglobin.
