# Supplementary material for: The Diversity of Mammalian Hemoproteins and Microbial Heme Scavengers Is Shaped by an Arms Race for Iron Piracy
Source: Front Immunol. 2018 Sep 11;9:2086. doi: 10.3389/fimmu.2018.02086 (PMC6142043; doi:10.3389/fimmu.2018.02086)
Supplement: Supplementary file 1 [file Table_1.PDF]

Supplementary Material

The diversity of mammalian hemoproteins and microbial heme scavengers is shaped by an arms race for iron piracy

Alessandra Mozzi*, Diego Forni, Mario Clerici, Rachele Cagliani, Manuela Sironi

*** Correspondence:** Alessandra Mozzi: alessandra.mozzi@bp.lnf.it

Supplementary Tables

Supplementary Table S1. List of mammalian species

Supplementary Table S1 . List of mammalian species

Order	*Scientific Name*	Common Name	Accession IDs	
			HBB	***HPX***
**Euarchontoglires**				
Primates	*Aotus nancymaae*	Nancy Ma's night monkey	XM_012456809.1	XM_012456768.1
	Callithrix jacchus	Marmoset	DP000589.1	XM_003734200.2
	Carlito syrichta	Philippine tarsier	-	XM_008050001.2
	Cebus capucinus	White-headed capuchin	XM_017507286.1	XM_017507146.1
	Cercocebus atys	Sooty mangabey	NW_012005601.1	XM_012036505.1
	Chlorocebus aethiops	Grivet	NT_166581.1	-
	Chlorocebus sabaeus	Green monkey	NM_001329918.1	XM_008008728.1
	Colobus angolensis	Sclater's Angola colobus	XM_011963601.1	XM_011954313.1
	Colobus guereza	Mantled guereza	NT_165846	-
	Gorilla gorilla	Gorilla	XM_019036164.1	XM_004050596.2
	Homo sapiens	Human	NM_000518	AH002827.2
	Macaca fascicularis	Crab-eating macaque	NM_001283367.1	XM_005578799.2
	Macaca mulatta	Rhesus macaque	NM_001164428.1	XM_001109797.3
	Macaca nemestrina	Southern pig-tailed macaque	XM_011718821.1	XM_011752986.1
	Mandrillus leucophaeus	Mandrill	XM_011975168.1	XM_011972504.1
	Microcebus murinus	Mouse lemur	-	XM_012786547.1
	Nasalis larvatus	Proboscis monkey	CM003003.1	-
	Nomascus leucogenys	Gibbon	ENSNLEG00000026827	XM_003254857.1
	Otolemur garnettii	Bushbaby	-	XM_003781033.2
	Pan paniscus	Bonobo	XM_003819029.2	XM_003819080.2
	Pan troglodytes	Chimpanzee	ENSPTRG00000040047	XM_508255.5
	Papio anubis	Olive baboon	ENSPANG00000004952	XM_003910295.2
	Plecturocebus moloch	Dusky titi monkey	ACA53486.1	-
	Pongo abelii	Orangutan	ENSPPYG00000003585	NM_001133595.1
	Propithecus coquereli	Coquerel's sifaka	-	XM_012654414.1
	Rhinopithecus bieti	Black snub-nosed monkey	-	XM_017893669.1
	Rhinopithecus roxellana	Snub-nosed monkey	XM_010363344.1	XM_010383525.1
	Saimiri boliviensis	Squirrel monkey	XM_003923369.2	XM_003919775.2
Dermoptera	*Galeopterus variegatus*	Sunda flying lemur	-	XM_008573689.1
Scadentia	*Tupaia chinensis*	Chinese tree shrew	-	XM_006161980.2
Lagomorpha	*Ochotona princeps*	Pika	-	XM_004590107.2
	Oryctolagus cuniculus	Rabbit	-	NM_001082760.1
Rodentia	*Castor canadensis*	North American beaver	-	XM_020179337.1
	Cavia porcellus	Guinea pig	-	XM_003465318.2
	Chinchilla lanigera	Chinchilla	-	XM_005380141.2
	Cricetulus griseus	Chinese hamster	-	XM_007607901.2
	Dipodomys ordii	Ord's kangaroo rat	-	XM_013035618.1
	Fukomys damarensis	Damaraland mole-rat	-	XM_010644253.2
	Heterocephalus glaber	Naked mole-rat	-	XM_004863391.3
	Ictidomys tridecemlineatus	Squirrel	-	XM_013365581.2
	Jaculus jaculus	Lesser Egyptian jerboa	-	XM_004650928.1
	Marmota marmota	Alpin marmot	-	XM_015477564.1
	Meriones unguiculatus	Mongolian gerbil	-	XM_021655088.1
	Mesocricetus auratus	Golden hamster	-	XM_005087437.3
	Microtus ochrogaster	Prairie vole	-	XM_005370219.1
	Mus caroli	Ryukyu mouse	-	XM_021168118.1
	Mus musculus	Mouse	-	NM_017371.2
	Mus pahari	Gairdner's shrewmouse	-	XM_021215187.1
	Nannospalax galili	Blind mole-rat	-	XM_008837388.2
	Octodon degus	Brush-tailed rat	-	XM_004642035.1
	Peromyscus maniculatus	Deer mouse	-	XM_006991732.2

	Rattus norvegicus	Rat	-	NM_053318.1
**Laurasiatheria**				
Artiodactyla	*Ammotragus lervia*	Barbary sheep	DQ352472.1	-
	Balaenoptera acutorostrata	Minke whale	-	XM_007172843.1
	Bison bison	American bison	XM_010832268.1	XM_010839138.1
	Bos grunniensis grunniensis	Domestic yak	DQ277007.1	-
	Bos indicus	Zebu	AB512644.1	XM_019975509.1
	Bos mutus	Wild yak	XM_005907921.1	XM_005907113.2
	Bos taurus	Cow	NM_173917.2	NM_001034612.2
	Bubalus bubalis	Water buffalo	AM886147.1	XM_006042108.1
	Camelus bactrianus	Bactrian camel	-	XM_010964963.1
	Camelus dromedarius	Dromedary	-	XM_010990544.1
	Camelus ferus	Wild bactrian camel	-	XM_014560092.1
	Capra aegagrus	Wild goat	JXYW01147663.1	-
	Capra hircus	Domestic goat	-	XM_005689766.3
	Lipotes vexillifer	Baiji	-	XM_007459815.1
	Odocoileus virginianus	White-tailed deer	-	XM_020896231.1
	Orcinus orca	Killer Whale	-	XM_004279283.1
	Ovis aries	Sheep	NM_001097648.1	XM_004016210.3
	Ovis aries musimon	Mouflon	NW_011944660.1	XM_012166032.2
	Ovis canadensis	Bighorn sheep	CP011900.1	-
	Pantholops hodgsonii	Tibetan antelope	-	XM_005960384.1
	Physeter catodon	Sperm whale	-	XM_007102995.1
	Sus scrofa	Pig	NM_001144841.1	NM_213953.2
	Tursiops truncatus	Dolphin	-	XM_019947849.1
	Vicugna pacos	Alpaca	-	XM_006203608.2
Carnivora	*Acinonyx jubatus*	Cheetah	-	XM_015079304.1
	Ailuropoda melanoleuca	Giant panda	NM_001304885.1	XM_019805688.1
	Canis lupus familiaris	Dog	XM_014122462.1	XM_848583.4
	Felis catus	Cat	XM_003992882.4	XM_003992929.4
	Mustela putorius furo	Ferret	XM_004779025.2	XM_004781168.2
	Neomonachus schauinslandi	Hawaiian monk seal	-	XM_021685705.1
	Odobenus rosmarus	Pacific walrus	-	XM_004413306.2
	Panthera leo	African lion	KR818803.1	-
	Panthera pardus	Leopard	-	XM_019456109.1
	Panthera tigris	Tiger	KR818802.1	XM_007083150.2
	Ursus maritimus	Polar bear	XM_008709612.1	XM_008711000.1
Chiroptera	*Eptesicus fuscus*	Big brown bat	-	XM_008159849.1
	Hipposideros armiger	Great roundleaf bat	-	XM_019632474.1
	Miniopterus natalensis	Natal long-fingered bat	-	XM_016217277.1
	Myotis brandtii	Brandt's bat	XM_005880400.2	XM_005886242.2
	Myotis davidii	David's myotis	-	XM_006770044.2
	Myotis lucifugus	Little brown bat	XM_006094539.2	XM_006105556.2
	Pteropus alecto	Black flying fox	XM_006915698.1	XM_006915661.1
	Pteropus vampyrus	Large flying fox	-	XM_011385180.1
	Rhinolophus ferrumequinum	Japanese greater horseshoe bat	DP000697.1	-
	Rhinolophus sinicus	Chinese rufous horseshoe bat	-	XM_019755870.1
	Rousettus aegyptiacus	Egyptian fruit bat	-	XM_016133515.1
Eulipotyphla	*Condylura cristata*	Star-nosed mole	-	XM_004683632.2
	Sorex araneus	Common shrew	-	XM_004621227.1
Perissodactyla	*Ceratotherium simum simum*	White rhinoceros	NW_004454203.1	-
	Equus asinus	Donkey	-	XM_014852123.1
	Equus caballus	Horse	NM_001164018.1	XM_005612117.2
	Equus przewalskii	Przewalski's Horse	XM_008523927.1	XM_008524127.1
	Erinaceus europaeus	European hedgehog	-	XM_007527815.2
Pholidota	*Manis javanica*	Malayan pangolins	XM_017676372.1	XM_017677857.1
**Afrotheria**				

Afrosoricida	*Chrysochloris asiatica*	Cape golden mole	-	XM_006870308.1
	Echinops telfairi	Lesser hedgehog tenrec	-	XM_004717126.1
Macroscelidea	*Elephantulus edwardii*	Cape elephant shrew	-	XM_006885632.1
Proboscidea	*Loxodonta africana*	African elephant	-	XM_003412219.1
Tubulidentata	*Orycteropus afer*	Aardvark	-	XM_007956436.1
