# Supplementary material for: The Diversity of Mammalian Hemoproteins and Microbial Heme Scavengers Is Shaped by an Arms Race for Iron Piracy
Source: Front Immunol. 2018 Sep 11;9:2086. doi: 10.3389/fimmu.2018.02086 (PMC6142043; doi:10.3389/fimmu.2018.02086)
Supplement: Supplementary file 2 [file Table_2.PDF]

Supplementary Material

The diversity of mammalian hemoproteins and microbial heme scavengers is shaped by an arms race for iron piracy

Alessandra Mozzi*, Diego Forni, Mario Clerici, Rachele Cagliani, Manuela Sironi

*** Correspondence:** Alessandra Mozzi: alessandra.mozzi@bp.lnf.it

Supplementary Tables

Supplementary Table S2. List of Trypanosome strains

Supplementary Table S2. List of *Trypanosome* strains

Organism	Strain/Type	Accession ID
Trypanosoma evansi	isolate ob106	JX143868eva.1
Trypanosoma equiperdum	isolate ob105	JX143867.1
Trypanosoma brucei gambiense	group 2 isolate HTAG15/5	JX143849.1
Trypanosoma brucei gambiense	group 2 isolate ob032	JX143850.1
Trypanosoma brucei gambiense	group 2 isolate ob146	JX143851.1
Trypanosoma brucei gambiense	group 2 isolate TH126	JX143853.1
Trypanosoma brucei gambiense	group 2 isolate ob151	JX143852.1
Trypanosoma brucei gambiense	group 1 isolate Tad	JX143848.1
Trypanosoma brucei gambiense	group 1 isolate ob202	JX143847.1
Trypanosoma brucei gambiense	group 1 isolate ob126	JX143845.1
Trypanosoma brucei gambiense	group 1 isolate ob186	JX143846.1
Trypanosoma brucei gambiense	group 1 isolate ob111	JX143844.1
Trypanosoma brucei gambiense	group 1 isolate ob107	JX143843.1
Trypanosoma brucei gambiense	group 1 isolate ob083	JX143842.1
Trypanosoma brucei gambiense	group 1 isolate ob080	JX143841.1
Trypanosoma brucei gambiense	group 1 isolate ob048	JX143840.1
Trypanosoma brucei gambiense	group 1 isolate ob007	JX143839.1
Trypanosoma brucei gambiense	group 1 isolate Fontem_strain_10	JX143838.1
Trypanosoma brucei gambiense	group 1 isolate Boula	JX143837.1
Trypanosoma brucei gambiense	group 1 isolate A004	JX143836.1
Trypanosoma brucei gambiense	group 1 isolate 1898	JX143835.1
Trypanosoma brucei gambiense	group 1 isolate 1829 (Aljo)	JX143834.1
Trypanosoma brucei rhodesiense	isolate ob156	JX143866.1
Trypanosoma brucei rhodesiense	isolate ob098	JX143865.1
Trypanosoma brucei rhodesiense	isolate ob096	JX143864.1
Trypanosoma brucei rhodesiense	isolate ob095	JX143863.1
Trypanosoma brucei rhodesiense	isolate ob093	JX143862.1
Trypanosoma brucei rhodesiense	isolate ob078	JX143861.1
Trypanosoma brucei rhodesiense	isolate ob066	JX143860.1
Trypanosoma brucei rhodesiense	isolate ob065	JX143859.1
Trypanosoma brucei rhodesiense	isolate ob056	JX143858.1
Trypanosoma brucei rhodesiense	isolate ob054	JX143857.1
Trypanosoma brucei rhodesiense	isolate ob021	JX143856.1
Trypanosoma brucei rhodesiense	isolate ob012	JX143855.1
Trypanosoma brucei rhodesiense	isolate ob018	JX143854.1
Trypanosoma brucei brucei	isolate ob155	JX143833.1
Trypanosoma brucei brucei	isolate ob154	JX143832.1
Trypanosoma brucei brucei	isolate SW3/87	JX143831.1
Trypanosoma brucei brucei	isolate TSW196	JX143830.1
Trypanosoma brucei brucei	isolate TSW187/78E	JX143829.1
Trypanosoma brucei brucei	isolate tRE087	JX143828.1
Trypanosoma brucei brucei	isolate PTAG130	JX143827.1
Trypanosoma brucei brucei	isolate PTAG_129	JX143826.1
Trypanosoma brucei brucei	isolate ob178	JX143825.1
Trypanosoma brucei brucei	isolate ob153	JX143824.1
Trypanosoma brucei brucei	isolate ob152	JX143823.1
Trypanosoma brucei brucei	isolate ob091	JX143822.1
Trypanosoma brucei brucei	isolate ob089	JX143821.1
Trypanosoma brucei brucei	isolate ob087	JX143820.1
Trypanosoma brucei brucei	isolate ob071	JX143819.1
Trypanosoma brucei brucei	isolate ob068	JX143818.1
Trypanosoma brucei brucei	isolate ob059	JX143817.1
Trypanosoma brucei brucei	isolate ob051	JX143816.1

Trypanosoma brucei brucei	isolate ob030	JX143815.1
Trypanosoma brucei brucei	isolate ob010	JX143814.1
Trypanosoma brucei brucei	isolate ob009	JX143813.1
Trypanosoma brucei brucei	isolate KP14 clone 1	JX143812.1
Trypanosoma brucei brucei	isolate KP13 clone 2	JX143811.1
Trypanosoma brucei brucei	isolate KP33 clone 8	JX143810.1
Trypanosoma brucei brucei	isolate KP14 clone 2	JX143809.1
Trypanosoma brucei brucei	isolate cp027	JX143808.1
Trypanosoma brucei brucei	isolate cp026	JX143807.1
Trypanosoma brucei brucei	isolate cp024	JX143806.1
Trypanosoma brucei brucei	isolate cp022	JX143805.1
Trypanosoma brucei brucei	isolate cp019	JX143804.1
Trypanosoma brucei brucei	isolate cp003	JX143803.1
Trypanosoma brucei brucei	isolate B8_18 clone B	JX143802.1
