# Supplementary material for: The Diversity of Mammalian Hemoproteins and Microbial Heme Scavengers Is Shaped by an Arms Race for Iron Piracy
Source: Front Immunol. 2018 Sep 11;9:2086. doi: 10.3389/fimmu.2018.02086 (PMC6142043; doi:10.3389/fimmu.2018.02086)
Supplement: Supplementary file 3 [file Table_3.PDF]

Supplementary Material

The diversity of mammalian hemoproteins and microbial heme scavengers is shaped by an arms race for iron piracy

Alessandra Mozzi*, Diego Forni, Mario Clerici, Rachele Cagliani, Manuela Sironi

*** Correspondence:** Alessandra Mozzi: alessandra.mozzi@bp.lnf.it

Supplementary Tables

Supplementary Table S3. List of *Staphylococcus* strains

Supplementary Table S3. **List of *Staphylococcus* strains**

**Organism/Strain**	**Assembly**	**Accession ID**
Staphylococcus argenteus BN75	GCA_001891145.1	NZ_CP015758.1
Staphylococcus argenteus MSHR1132	GCA_000236925.1	NC_016941.1
Staphylococcus aureus 04-02981	GCA_000025145.2	NC_017340.1
Staphylococcus aureus 08-02119	GCA_001656045.1	NZ_CP015645.1
Staphylococcus aureus 08-02300	GCA_001656075.1	NZ_CP015646.1
Staphylococcus aureus 08BA02176	GCA_000296595.1	NC_018608.1
Staphylococcus aureus 144_S7	GCA_000934245.1	CP010943.1
Staphylococcus aureus 25b_MRSA	GCA_000815205.1	NZ_CP010299.1
Staphylococcus aureus 71A_S11	GCA_000934185.1	CP010940.1
Staphylococcus aureus 93b_S9	GCA_000934925.1	CP010952.1
Staphylococcus aureus AUS0325	GCA_900096745.1	NZ_LT615218.1
Staphylococcus aureus BB155	GCA_900004855.1	NZ_LN854556.1
Staphylococcus aureus CA-347	GCA_000412775.1	NC_021554.1
Staphylococcus aureus FCFHV36	GCA_000969225.1	NZ_CP011147.1
Staphylococcus aureus FDA209P	GCA_001548295.1	NZ_AP014942.1
Staphylococcus aureus FDAARGOS_159	GCA_001558795.1	NZ_CP014064.1
Staphylococcus aureus LGA251	GCA_000237265.1	NC_017349.1
Staphylococcus aureus MCRF184	GCA_001594205.1	NZ_CP014791.1
Staphylococcus aureus MS4	GCA_001456215.1	NZ_CP009828.1
Staphylococcus aureus NCCP14558	GCA_001640905.1	CP013953.1
Staphylococcus aureus NZAK3	GCA_900017775.1	NZ_LT009690.1
Staphylococcus aureus OC8	GCA_002355355.1	NZ_AP017377.1
Staphylococcus aureus Reynolds	--	KJ001295.1
Staphylococcus aureus RF122	GCA_000009005.1	NC_007622.1
Staphylococcus aureus RIVM1295	GCA_001465635.1	CP013616.1
Staphylococcus aureus RIVM3897	GCA_001465755.1	CP013621.1
Staphylococcus aureus RIVM6519	GCA_001618305.1	NZ_CP015173.1
Staphylococcus aureus RKI4	GCA_001027045.1	NZ_CP011528.1
Staphylococcus aureus SA17_S6	GCA_000934205.1	CP010941.1
Staphylococcus aureus ST20130940	GCA_001611325.1	NZ_CP012979.1
Staphylococcus aureus ST20130943	GCA_001611385.1	NZ_CP012974.1
Staphylococcus aureus subsp. aureus 11819-97	GCA_000239235.1	NC_017351.1
Staphylococcus aureus subsp. aureus 6850	GCA_000462955.1	NC_022222.1
Staphylococcus aureus subsp. aureus CN1	GCA_000463055.1	NC_022226.1
Staphylococcus aureus subsp. aureus ED133	GCA_000210315.1	NC_017337.1
Staphylococcus aureus subsp. aureus ED98	GCA_000024585.1	NC_013450.1
Staphylococcus aureus subsp. aureus MSSA476	GCA_000011525.1	NC_002953.3
Staphylococcus aureus subsp. aureus ST398	GCA_000009585.1	NC_017333.1
Staphylococcus aureus subsp. aureus TW20	GCA_000027045.1	NC_017331.1
Staphylococcus aureus subsp. aureus USA300_TCH1516	GCA_000017085.1	NC_010079.1
Staphylococcus aureus subsp. aureus VC40	GCA_000245495.1	NC_016912.1
Staphylococcus aureus TCH60	GCA_000159535.2	NC_017342.1
Staphylococcus aureus V2200	GCA_001046095.2	NZ_CP007657.1
Staphylococcus aureus V605	GCA_001641045.1	CP013959.1
Staphylococcus aureus XN108	GCA_000709475.1	CP007447.1
Staphylococcus aureus XQ	GCA_001444345.1	NZ_CP013137.1
