# Supplementary material for: The Diversity of Mammalian Hemoproteins and Microbial Heme Scavengers Is Shaped by an Arms Race for Iron Piracy
Source: Front Immunol. 2018 Sep 11;9:2086. doi: 10.3389/fimmu.2018.02086 (PMC6142043; doi:10.3389/fimmu.2018.02086)
Supplement: Supplementary file 4 [file Table_4.PDF]

Supplementary Material

The diversity of mammalian hemoproteins and microbial heme scavengers is shaped by an arms race for iron piracy

Alessandra Mozzi*, Diego Forni, Mario Clerici, Rachele Cagliani, Manuela Sironi

*** Correspondence:** Alessandra Mozzi: alessandra.mozzi@bp.lnf.it

Supplementary Tables

Supplementary Table S4. List of *Neisseria meningitidis* strains

Supplementary Table S4. **List of *Neisseria meningitidis* strains**

Organism/Strain	Assembly	Accession ID
Neisseria meningitidis 2000063	GCA_000386465.2	APTA01
Neisseria meningitidis 2000080	GCA_000386145.2	APSI01
Neisseria meningitidis 2000081	GCA_000386925.2	APUA01
Neisseria meningitidis 2000175	GCA_000387025.2	APUF01
Neisseria meningitidis 2001001	GCA_000386945.2	APUB01
Neisseria meningitidis 2001072	GCA_000386885.2	APTY01
Neisseria meningitidis 2001073	GCA_000386905.2	APTZ01
Neisseria meningitidis 2001212	GCA_000328065.2	ANSF01
Neisseria meningitidis 2001213	GCA_000386985.2	APUD01
Neisseria meningitidis 2002004	GCA_000387085.2	APUJ01
Neisseria meningitidis 2002007	GCA_000386485.2	APTB01
Neisseria meningitidis 2003022	GCA_000392695.2	APSZ01
Neisseria meningitidis 2003051	GCA_000387145.2	APUP01
Neisseria meningitidis 2004032	GCA_000386965.2	APUC01
Neisseria meningitidis 2004085	GCA_000386445.2	APSY01
Neisseria meningitidis 2004090	GCA_000328105.2	ANRW01
Neisseria meningitidis 2004264	GCA_000387005.2	APUE01
Neisseria meningitidis 2005040	GCA_000387065.2	APUI01
Neisseria meningitidis 2005079	GCA_000387045.2	APUG01
Neisseria meningitidis 2007056	GCA_000327725.2	ANSE01
Neisseria meningitidis 2007461	GCA_000413195.2	APUK01
Neisseria meningitidis 331401	GCA_001750825.1	NZ_CP012694.1
Neisseria meningitidis 510612	GCA_000626595.1	NZ_CP007524.1
Neisseria meningitidis 61103	GCA_000327765.2	ANRZ01
Neisseria meningitidis 61106	GCA_000386285.2	APSQ01
Neisseria meningitidis 63006	GCA_000327545.2	ANSI01
Neisseria meningitidis 63023	GCA_000386265.2	APSP01
Neisseria meningitidis 63041	GCA_000327665.2	ANRF01
Neisseria meningitidis 63049	GCA_000327805.2	ANRV01
Neisseria meningitidis 64182	GCA_000386345.2	APST01
Neisseria meningitidis 65012	GCA_000386325.2	APSS01
Neisseria meningitidis 65014	GCA_000327785.2	ANRY01
Neisseria meningitidis 68094	GCA_000328245.2	ANRB01
Neisseria meningitidis 69096	GCA_000327745.2	ANSB01
Neisseria meningitidis 69100	GCA_000386245.2	APSN01
Neisseria meningitidis 69155	GCA_000386085.2	APSF01
Neisseria meningitidis 69166	GCA_000293365.1	ALXS01
Neisseria meningitidis 69176	GCA_000386105.2	APSG01
Neisseria meningitidis 70012	GCA_000328225.2	ANRE01
Neisseria meningitidis 70021	GCA_000386125.2	APSH01
Neisseria meningitidis 70030	GCA_000328025.2	ANSH01
Neisseria meningitidis 70082	GCA_000386305.2	APSR01
Neisseria meningitidis 73696	GCA_000386745.2	APTR01
Neisseria meningitidis 73704	GCA_000386765.2	APTS01
Neisseria meningitidis 75643	GCA_000386205.2	APSL01
Neisseria meningitidis 75689	GCA_000386225.2	APSM01
Neisseria meningitidis 80179	GCA_000293425.1	ALXV01
Neisseria meningitidis 87255	GCA_000327705.2	ANQZ01
Neisseria meningitidis 88050	GCA_000327985.2	ANRD01
Neisseria meningitidis 92045	GCA_000293445.1	ALXW01
Neisseria meningitidis 94018	GCA_000386185.2	APSK01
Neisseria meningitidis 96023	GCA_000327605.2	ANRX01
Neisseria meningitidis 96024	GCA_000386385.2	APSV01

Neisseria meningitidis 96060	GCA_000386165.2	APSJ01
Neisseria meningitidis 961-5945	GCA_000191325.2	AEQK01
Neisseria meningitidis 97008	GCA_000386405.2	APSW01
Neisseria meningitidis 97018	GCA_000392355.1	APSO01
Neisseria meningitidis 97020	GCA_000327585.2	ANSA01
Neisseria meningitidis 97027	GCA_000386365.2	APSU01
Neisseria meningitidis 98005	GCA_000386425.2	APSX01
Neisseria meningitidis 98080	GCA_000328005.2	ANRA01
Neisseria meningitidis alpha704	GCA_000304435.1	CAJS01
Neisseria meningitidis alpha710	GCA_000152165.1	NC_017505.1
Neisseria meningitidis ATCC 13091	GCA_000146655.1	AEEF01
Neisseria meningitidis B6116/77	GCA_001029815.1	NZ_CP007667.1
Neisseria meningitidis CU385	GCA_000191305.2	AEQJ01
Neisseria meningitidis DE10444	GCA_001697125.1	NZ_CP012392.1
Neisseria meningitidis DE8555	GCA_001697165.1	NZ_CP012393.1
Neisseria meningitidis ES14902	GCA_000191285.2	AEQI01
Neisseria meningitidis FAM18	GCA_000009465.1	NC_008767.1
Neisseria meningitidis G2136	GCA_000191425.1	NC_017513.1
Neisseria meningitidis L91543	GCA_000787195.2	NZ_CP016684.1
Neisseria meningitidis LNP27256	GCA_000464995.1	AVOU01
Neisseria meningitidis M01-240013	GCA_000191345.2	AEQL01
Neisseria meningitidis M07149	GCA_001697425.1	NZ_CP016650.1
Neisseria meningitidis M07161	GCA_001697925.1	NZ_CP016675.1
Neisseria meningitidis M07162	GCA_001698045.1	NZ_CP016644.1
Neisseria meningitidis M08000	GCA_001698065.1	NZ_CP016681.1
Neisseria meningitidis M08001	GCA_001697465.1	NZ_CP016652.1
Neisseria meningitidis M09261	GCA_001697725.1	NZ_CP016665.1
Neisseria meningitidis M09293	GCA_001697385.1	NZ_CP016648.1
Neisseria meningitidis M10208	GCA_000800415.1	NZ_CP009422.1
Neisseria meningitidis M12752	GCA_001697325.1	NZ_CP016645.1
Neisseria meningitidis M13399	GCA_000191245.2	AEQG01
Neisseria meningitidis M22160	GCA_001697905.1	NZ_CP016674.1
Neisseria meningitidis M22189	GCA_001697405.1	NZ_CP016649.1
Neisseria meningitidis M22191	GCA_001698105.1	NZ_CP016683.1
Neisseria meningitidis M22718	GCA_001697305.1	NZ_CP016627.1
Neisseria meningitidis M22722	GCA_001697685.1	NZ_CP016663.1
Neisseria meningitidis M22740	GCA_001698005.1	NZ_CP016679.1
Neisseria meningitidis M22745	GCA_001697565.1	NZ_CP016657.1
Neisseria meningitidis M22748	GCA_001697485.1	NZ_CP016653.1
Neisseria meningitidis M22759	GCA_001697805.1	NZ_CP016669.1
Neisseria meningitidis M22769	GCA_001697545.1	NZ_CP016656.1
Neisseria meningitidis M22772	GCA_001697525.1	NZ_CP016655.1
Neisseria meningitidis M22783	GCA_001697845.1	NZ_CP016671.1
Neisseria meningitidis M22801	GCA_001697605.1	NZ_CP016659.1
Neisseria meningitidis M22804	GCA_001697625.1	NZ_CP016660.1
Neisseria meningitidis M22809	GCA_001697365.1	NZ_CP016647.1
Neisseria meningitidis M22811	GCA_001697505.1	NZ_CP016654.1
Neisseria meningitidis M22819	GCA_001697345.1	NZ_CP016646.1
Neisseria meningitidis M22822	GCA_001698025.1	NZ_CP016680.1
Neisseria meningitidis M22828	GCA_001697865.1	NZ_CP016672.1
Neisseria meningitidis M23413	GCA_001697665.1	NZ_CP016662.1
Neisseria meningitidis M24705	GCA_001698085.1	NZ_CP016682.1
Neisseria meningitidis M24730	GCA_001697585.1	NZ_CP016658.1
Neisseria meningitidis M25070	GCA_001697705.1	NZ_CP016664.1
Neisseria meningitidis M25087	GCA_001697825.1	NZ_CP016670.1
Neisseria meningitidis M25419	GCA_001697985.1	NZ_CP016678.1

Neisseria meningitidis M25438	GCA_001697645.1	NZ_CP016661.1
Neisseria meningitidis M25456	GCA_001697965.1	NZ_CP016677.1
Neisseria meningitidis M25459	GCA_001697885.1	NZ_CP016673.1
Neisseria meningitidis M25462	GCA_001697745.1	NZ_CP016666.1
Neisseria meningitidis M25472	GCA_001697785.1	NZ_CP016668.1
Neisseria meningitidis M25474	GCA_001697445.1	NZ_CP016651.1
Neisseria meningitidis M25476	GCA_001697945.1	NZ_CP016676.1
Neisseria meningitidis M27559	GCA_001697765.1	NZ_CP016667.1
Neisseria meningitidis M6190	GCA_000191225.2	AEQF01
Neisseria meningitidis M7089	GCA_000328165.2	ANRN01
Neisseria meningitidis M7124	GCA_000800275.1	NZ_CP009419.1
Neisseria meningitidis NM003	GCA_000448125.1	AVNQ01
Neisseria meningitidis NM045	GCA_000448085.1	AVNO01
Neisseria meningitidis NM126	GCA_000328145.2	ANRQ01
Neisseria meningitidis NM133	GCA_000386565.2	APTF01
Neisseria meningitidis NM134	GCA_000413215.2	APUL01
Neisseria meningitidis NM140	GCA_000293305.1	ALXP01
Neisseria meningitidis NM1482	GCA_000392715.2	APTP01
Neisseria meningitidis NM1495	GCA_000386725.2	APTQ01
Neisseria meningitidis NM165	GCA_000387285.2	APVC01
Neisseria meningitidis NM174	GCA_000327885.2	ANRP01
Neisseria meningitidis NM183	GCA_000293325.1	ALXQ01
Neisseria meningitidis NM220	GCA_000242755.2	AGRR01
Neisseria meningitidis NM23	GCA_000387325.2	APTG01
Neisseria meningitidis NM233	GCA_000242735.2	AGRQ01
Neisseria meningitidis NM255	GCA_000293285.1	ALXO01
Neisseria meningitidis NM2657	GCA_000293465.1	ALXX01
Neisseria meningitidis NM27	GCA_000392735.2	APUZ01
Neisseria meningitidis NM271	GCA_000387165.2	APUQ01
Neisseria meningitidis NM2781	GCA_000293345.1	ALXR01
Neisseria meningitidis NM2795	GCA_000293625.1	ALXY01
Neisseria meningitidis NM2866	GCA_000448245.1	AVNW01
Neisseria meningitidis NM3001	GCA_000293665.1	ALYA01
Neisseria meningitidis NM3042	GCA_000386065.2	APUT01
Neisseria meningitidis NM313	GCA_000387225.2	APTO01
Neisseria meningitidis NM3131	GCA_000386605.2	APUV01
Neisseria meningitidis NM3139	GCA_000448065.1	AVNN01
Neisseria meningitidis NM3144	GCA_000386625.2	APUW01
Neisseria meningitidis NM3147	GCA_000386865.2	APTX01
Neisseria meningitidis NM3158	GCA_000386645.2	APUX01
Neisseria meningitidis NM3164	GCA_000386665.2	APUY01
Neisseria meningitidis NM32	GCA_000387345.2	APTH01
Neisseria meningitidis NM3222	GCA_000386585.2	APUU01
Neisseria meningitidis NM3223	GCA_000387305.2	APVD01
Neisseria meningitidis NM35	GCA_000387365.2	APTI01
Neisseria meningitidis NM36	GCA_000387385.2	APTJ01
Neisseria meningitidis NM3642	GCA_000327565.2	ANSD01
Neisseria meningitidis NM3652	GCA_000328085.2	ANSC01
Neisseria meningitidis NM3682	GCA_000800315.1	NZ_CP009420.1
Neisseria meningitidis NM3683	GCA_000800355.1	NZ_CP009421.1
Neisseria meningitidis NM3686	GCA_000800235.1	NZ_CP009418.1
Neisseria meningitidis NM43	GCA_000386705.2	APTK01
Neisseria meningitidis NM518	GCA_000448185.1	AVNT01
Neisseria meningitidis NM576	GCA_000293385.1	ALXT01
Neisseria meningitidis NM586	GCA_000327925.2	ANRL01
Neisseria meningitidis NM604	GCA_000386505.2	APTC01

Neisseria meningitidis NM606	GCA_000386525.2	APTD01
Neisseria meningitidis NM607	GCA_000386545.2	APTE01
Neisseria meningitidis NM762	GCA_000327905.2	ANRM01
Neisseria meningitidis NM80	GCA_000387265.2	APVB01
Neisseria meningitidis NM82	GCA_000387185.2	APTL01
Neisseria meningitidis NM90	GCA_000386045.2	APUS01
Neisseria meningitidis NM94	GCA_000387205.2	APTM01
Neisseria meningitidis NM95	GCA_000387245.2	APTN01
Neisseria meningitidis WUE 2121	GCA_001697205.1	NZ_CP012394.1
Neisseria meningitidis WUE 2594	GCA_000253215.1	NC_017512.1
Neisseria meningitidis Z2491	GCA_000009105.1	NC_003116.1
