# Supplementary material for: The Diversity of Mammalian Hemoproteins and Microbial Heme Scavengers Is Shaped by an Arms Race for Iron Piracy
Source: Front Immunol. 2018 Sep 11;9:2086. doi: 10.3389/fimmu.2018.02086 (PMC6142043; doi:10.3389/fimmu.2018.02086)
Supplement: Supplementary file 5 [file Table_5.PDF]

Supplementary Material

The diversity of mammalian hemoproteins and microbial heme scavengers is shaped by an arms race for iron piracy

Alessandra Mozzi*, Diego Forni, Mario Clerici, Rachele Cagliani, Manuela Sironi

*** Correspondence:** Alessandra Mozzi: alessandra.mozzi@bp.lnf.it

Supplementary Tables

Supplementary Table S5. List of *Neisseria gonorrhoeae* strains

Supplementary Table S5. List of *Neisseria gonorrhoeae* strains

Organism/Strain	Assembly	Accession ID
Neisseria gonorrhoeae 1291	GCA_000156755.1	ABZF01
Neisseria gonorrhoeae 32867	GCA_001661085.1	NZ_CP016015.1
Neisseria gonorrhoeae 34530	GCA_001661115.1	NZ_CP016016.1
Neisseria gonorrhoeae 35/02	GCA_001047275.1	NZ_CP012028.1
Neisseria gonorrhoeae ALB_2011_01-02	GCA_001025905.1	ATQE01
Neisseria gonorrhoeae ALB_2011_03_03	GCA_001026275.1	ATQB01
Neisseria gonorrhoeae ALB_2011_04_03	GCA_001025915.1	ATQC01
Neisseria gonorrhoeae ATCC 49226	GCA_001997645.1	MTGL01
Neisseria gonorrhoeae ATL_2011_01_03	GCA_001025585.1	ATQD01
Neisseria gonorrhoeae ATL_2011_01_05	GCA_001025945.1	ATQF01
Neisseria gonorrhoeae ATL_2011_01_08	GCA_001025955.1	ATQG01
Neisseria gonorrhoeae ATL_2011_01_17	GCA_001026255.1	ATQH01
Neisseria gonorrhoeae ATL_2011_01-21	GCA_001025595.1	ATQI01
Neisseria gonorrhoeae ATL_2011_01-25	GCA_001025985.1	ATQJ01
Neisseria gonorrhoeae ATL_2011_05-08	GCA_001026305.1	ATQK01
Neisseria gonorrhoeae ATL_2011_05-13	GCA_001025615.1	ATQL01
Neisseria gonorrhoeae CH811	GCA_001026555.1	ATKL01
Neisseria gonorrhoeae DGI18	GCA_000156795.1	ABZH01
Neisseria gonorrhoeae DGI2	GCA_000159935.1	ACIG01
Neisseria gonorrhoeae F62	GCA_000163535.1	ADAA01
Neisseria gonorrhoeae FA 1090	GCA_000006845.1	NC_002946.2
Neisseria gonorrhoeae FA19	GCA_001047225.1	NZ_CP012026.1
Neisseria gonorrhoeae FDAARGOS_204	GCA_002073535.1	NZ_CP020415.1
Neisseria gonorrhoeae FDAARGOS_205	GCA_002073615.1	MBCP01
Neisseria gonorrhoeae FDAARGOS_207	GCA_002073655.1	NZ_CP020419.1
Neisseria gonorrhoeae GC1-182	GCA_001026545.1	ATPD01
Neisseria gonorrhoeae GCGS001	GCA_001158945.1	CQOO01
Neisseria gonorrhoeae GCGS002	GCA_001124565.1	CQLO01
Neisseria gonorrhoeae GCGS004	GCA_001103845.1	CQJL01
Neisseria gonorrhoeae GCGS005	GCA_001084085.1	CQOF01
Neisseria gonorrhoeae GCGS006	GCA_001128365.1	CQLM01
Neisseria gonorrhoeae GCGS009	GCA_001099005.1	CQJO01
Neisseria gonorrhoeae GCGS017	GCA_001131645.1	CQIC01
Neisseria gonorrhoeae GCGS019	GCA_001162285.1	CQOE01
Neisseria gonorrhoeae GCGS022	GCA_001103565.1	CQIP01
Neisseria gonorrhoeae GCGS025	GCA_001096365.1	CQKE01
Neisseria gonorrhoeae GCGS027	GCA_001116825.1	CQNY01
Neisseria gonorrhoeae GCGS029	GCA_001154265.1	CQNA01
Neisseria gonorrhoeae GCGS031	GCA_001161025.1	CQOY01
Neisseria gonorrhoeae GCGS033	GCA_001355095.1	CFMC01
Neisseria gonorrhoeae GCGS034	GCA_001146625.1	CQLT01
Neisseria gonorrhoeae GCGS035	GCA_001088485.1	CQKA01
Neisseria gonorrhoeae GCGS036	GCA_001130625.1	CQLA01
Neisseria gonorrhoeae GCGS040	GCA_001083925.1	CQLY01
Neisseria gonorrhoeae GCGS041	GCA_001171665.1	CQHM01
Neisseria gonorrhoeae GCGS058	GCA_001150905.1	CQKG01
Neisseria gonorrhoeae GCGS060	GCA_001162645.1	CQLX01
Neisseria gonorrhoeae GCGS077	GCA_001145045.1	CQNB01
Neisseria gonorrhoeae GCGS083	GCA_001147965.1	CQKQ01
Neisseria gonorrhoeae GCGS084	GCA_001103805.1	CQNZ01
Neisseria gonorrhoeae GCGS086	GCA_001106145.1	CQMJ01
Neisseria gonorrhoeae GCGS089	GCA_001168105.1	CQID01
Neisseria gonorrhoeae GCGS099	GCA_001165845.1	CQMR01

Neisseria gonorrhoeae GCGS104	GCA_001354755.1	CFMA01
Neisseria gonorrhoeae GCGS109	GCA_001172285.1	CQKH01
Neisseria gonorrhoeae GCGS113	GCA_001164185.1	CQNI01
Neisseria gonorrhoeae GCGS115	GCA_001100365.1	CQLD01
Neisseria gonorrhoeae GCGS116	GCA_001145225.1	CQOD01
Neisseria gonorrhoeae GCGS117	GCA_001095225.1	CQKP01
Neisseria gonorrhoeae GCGS118	GCA_001131965.1	CQNL01
Neisseria gonorrhoeae GCGS120	GCA_001355175.1	CIFS01
Neisseria gonorrhoeae GCGS126	GCA_001129885.1	CQJX01
Neisseria gonorrhoeae GCGS128	GCA_001112845.1	CQKW01
Neisseria gonorrhoeae GCGS142	GCA_001156005.1	CQMS01
Neisseria gonorrhoeae GCGS155	GCA_001355015.1	CHDW01
Neisseria gonorrhoeae GCGS163	GCA_001116105.1	CQKY01
Neisseria gonorrhoeae GCGS165	GCA_001151205.1	CQJQ01
Neisseria gonorrhoeae GCGS168	GCA_001355195.1	CGDD01
Neisseria gonorrhoeae GCGS170	GCA_001355335.1	CFLX01
Neisseria gonorrhoeae GCGS171	GCA_001102165.1	CQLF01
Neisseria gonorrhoeae GCGS174	GCA_001170125.1	CQOT01
Neisseria gonorrhoeae GCGS176	GCA_001143005.1	CQNJ01
Neisseria gonorrhoeae GCGS180	GCA_001133405.1	CQOW01
Neisseria gonorrhoeae GCGS188	GCA_001171845.1	CQLV01
Neisseria gonorrhoeae GCGS189	GCA_001161245.1	CQOH01
Neisseria gonorrhoeae GCGS190	GCA_001355155.1	CFSG01
Neisseria gonorrhoeae GCGS191	GCA_001129585.1	CQLW01
Neisseria gonorrhoeae GCGS202	GCA_001085725.1	CQJM01
Neisseria gonorrhoeae GCGS203	GCA_001156545.1	CQKM01
Neisseria gonorrhoeae GCGS205	GCA_001120385.1	CQJU01
Neisseria gonorrhoeae GCGS208	GCA_001125025.1	CQKT01
Neisseria gonorrhoeae GCGS210	GCA_001088845.1	CQJI01
Neisseria gonorrhoeae GCGS215	GCA_001087905.1	CQME01
Neisseria gonorrhoeae GCGS218	GCA_001103185.1	CQMZ01
Neisseria gonorrhoeae GCGS222	GCA_001151425.1	CQLN01
Neisseria gonorrhoeae GCGS227	GCA_001099685.1	CQKN01
Neisseria gonorrhoeae GCGS228	GCA_001099505.1	CQMA01
Neisseria gonorrhoeae GCGS229	GCA_001319165.1	CHZL01
Neisseria gonorrhoeae GCGS233	GCA_001132085.1	CQIJ01
Neisseria gonorrhoeae GCGS234	GCA_001117665.1	CQKL01
Neisseria gonorrhoeae GCGS235	GCA_001167285.1	CQIK01
Neisseria gonorrhoeae GCGS237	GCA_001156065.1	CQHY01
Neisseria gonorrhoeae GCGS239	GCA_001132185.1	CQKF01
Neisseria gonorrhoeae H15_353	GCA_001563755.1	LOIB01
Neisseria gonorrhoeae m07.05	GCA_000695445.1	JFBB01
Neisseria gonorrhoeae MIA_2011_02_02	GCA_001025645.1	ATQM01
Neisseria gonorrhoeae MIA_2011_03-09	GCA_001026315.1	ATQN01
Neisseria gonorrhoeae MIA_2011_03-10	GCA_001025995.1	ATQO01
Neisseria gonorrhoeae MIA_2011_05-10	GCA_001026335.1	ATQP01
Neisseria gonorrhoeae MIA_2011_05-15	GCA_001025655.1	ATQQ01
Neisseria gonorrhoeae MIA_2011_05-16	GCA_001026025.1	ATQR01
Neisseria gonorrhoeae MS11	GCA_000156855.2	NC_022240.1
Neisseria gonorrhoeae MU_NG1	GCA_001026385.1	ATQV01
Neisseria gonorrhoeae MU_NG12	GCA_001026105.1	ATRC01
Neisseria gonorrhoeae MU_NG14	GCA_001026465.1	ATRD01
Neisseria gonorrhoeae MU_NG15	GCA_001025715.1	ATRE01
Neisseria gonorrhoeae MU_NG17	GCA_001026565.1	ATRF01
Neisseria gonorrhoeae MU_NG19	GCA_001026625.1	ATRH01
Neisseria gonorrhoeae MU_NG20	GCA_001026655.1	ATRI01

Neisseria gonorrhoeae MU_NG21	GCA_001026115.1	ATRJ01
Neisseria gonorrhoeae MU_NG23	GCA_001026485.1	ATRK01
Neisseria gonorrhoeae MU_NG25	GCA_001026645.1	ATRL01
Neisseria gonorrhoeae MU_NG26	GCA_001026615.1	ATRM01
Neisseria gonorrhoeae MU_NG3	GCA_001026395.1	ATQW01
Neisseria gonorrhoeae MU_NG4	GCA_001025705.1	ATQX01
Neisseria gonorrhoeae MU_NG5	GCA_001026065.1	ATQY01
Neisseria gonorrhoeae MU_NG6	GCA_001026415.1	ATQZ01
Neisseria gonorrhoeae MU_NG8	GCA_001026085.1	ATRA01
Neisseria gonorrhoeae MU_NG9	GCA_001026405.1	ATRB01
Neisseria gonorrhoeae NCCP11945	GCA_000020105.1	NC_011035.1
Neisseria gonorrhoeae NCTC13798	GCA_900186875.1	NZ_LT906440.1
Neisseria gonorrhoeae NCTC13799	GCA_900186935.1	NZ_LT906437.1
Neisseria gonorrhoeae NCTC13800	GCA_900186915.1	NZ_LT906472.1
Neisseria gonorrhoeae NG_869	GCA_001039435.1	LFJW01
Neisseria gonorrhoeae NGSJH1	GCA_002080955.1	NAGH01
Neisseria gonorrhoeae NGSJH10	GCA_002080825.1	NAGO01
Neisseria gonorrhoeae NGSJH11	GCA_002080855.1	NAGP01
Neisseria gonorrhoeae NGSJH2	GCA_002080715.1	NAGI01
Neisseria gonorrhoeae NGSJH4	GCA_002080745.1	NAGJ01
Neisseria gonorrhoeae NGSJH5	GCA_002080775.1	NAGK01
Neisseria gonorrhoeae NOR_2011_03-06	GCA_001025685.1	ATQS01
Neisseria gonorrhoeae NYC_2011_05_07	GCA_001026345.1	ATQT01
Neisseria gonorrhoeae NYC_2011_05_13	GCA_001026035.1	ATQU01
Neisseria gonorrhoeae PID1	GCA_000156895.1	ABZM01
Neisseria gonorrhoeae PID18	GCA_000156875.1	ABZL01
Neisseria gonorrhoeae PID24-1	GCA_000156915.1	ABZN01
Neisseria gonorrhoeae PID332	GCA_000156935.1	ABZO01
Neisseria gonorrhoeae SK-92-679	GCA_000156955.1	ABZP01
Neisseria gonorrhoeae SK-93-1035	GCA_000156975.1	ABZQ01
Neisseria gonorrhoeae SK12684	GCA_001026535.1	ATPK01
Neisseria gonorrhoeae SK14515	GCA_001025485.1	ATPM01
Neisseria gonorrhoeae SK15454	GCA_001025805.1	ATPN01
Neisseria gonorrhoeae SK16942	GCA_001025835.1	ATPP01
Neisseria gonorrhoeae SK17973	GCA_001025475.1	ATPQ01
Neisseria gonorrhoeae SK1902	GCA_001026145.1	ATPF01
Neisseria gonorrhoeae SK22871	GCA_001025525.1	ATPR01
Neisseria gonorrhoeae SK23020	GCA_001025535.1	ATPS01
Neisseria gonorrhoeae SK28355	GCA_001026185.1	ATPU01
Neisseria gonorrhoeae SK29344	GCA_001026175.1	ATPV01
Neisseria gonorrhoeae SK29471	GCA_001025865.1	ATPW01
Neisseria gonorrhoeae SK32402	GCA_001025545.1	ATPX01
Neisseria gonorrhoeae SK33414	GCA_001025875.1	ATPY01
Neisseria gonorrhoeae SK36809	GCA_001026225.1	ATPZ01
Neisseria gonorrhoeae SK39420	GCA_001026245.1	ATQA01
Neisseria gonorrhoeae SK6987	GCA_001026165.1	ATPG01
Neisseria gonorrhoeae SK708	GCA_001025745.1	ATPE01
Neisseria gonorrhoeae SK7461	GCA_001025465.1	ATPH01
Neisseria gonorrhoeae SK7842	GCA_001025765.1	ATPI01
Neisseria gonorrhoeae SK8976	GCA_001025775.1	ATPJ01
Neisseria gonorrhoeae USO_DK11-24	GCA_001859135.1	MBBI01
Neisseria gonorrhoeae USO_DK12-21	GCA_001859405.1	MBBV01
Neisseria gonorrhoeae USO_DK12-38	GCA_001859425.1	MBBW01
Neisseria gonorrhoeae USO_G09-145	GCA_001858745.1	MBAO01
Neisseria gonorrhoeae USO_GC3828	GCA_001859565.1	MBCD01
Neisseria gonorrhoeae USO_GC3831	GCA_001859585.1	MBCE01

Neisseria gonorrhoeae USO_GC3868	GCA_001859705.1	MBCK01
Neisseria gonorrhoeae USO_GR13-015	GCA_001859945.1	MBCW01
Neisseria gonorrhoeae USO_IE11027	GCA_001859185.1	MBBK01
Neisseria gonorrhoeae USO_IE11068	GCA_001859205.1	MBBL01
Neisseria gonorrhoeae USO_IE12-079	GCA_001859545.1	MBCC01
Neisseria gonorrhoeae USO_NL09-008	GCA_001858695.1	MBAM01
Neisseria gonorrhoeae USO_NL10-083	GCA_001859005.1	MBBB01
Neisseria gonorrhoeae USO_NO10-026	GCA_001858855.1	MBAU01
Neisseria gonorrhoeae USO_SI10-010	GCA_001858935.1	MBAY01
Neisseria gonorrhoeae USO_SI12-044	GCA_001859835.1	MBCR01
Neisseria gonorrhoeae USO_SP09-062	GCA_001858665.1	MBAL01
Neisseria gonorrhoeae USO_UK121470	GCA_001859875.1	MBCT01
