# Supplementary material for: The Diversity of Mammalian Hemoproteins and Microbial Heme Scavengers Is Shaped by an Arms Race for Iron Piracy
Source: Front Immunol. 2018 Sep 11;9:2086. doi: 10.3389/fimmu.2018.02086 (PMC6142043; doi:10.3389/fimmu.2018.02086)
Supplement: Supplementary file 6 [file Table_6.PDF]

Supplementary Material

The diversity of mammalian hemoproteins and microbial heme scavengers is shaped by an arms race for iron piracy

Alessandra Mozzi*, Diego Forni, Mario Clerici, Rachele Cagliani, Manuela Sironi

*** Correspondence:** Alessandra Mozzi: alessandra.mozzi@bp.lnf.it

Supplementary Tables

Supplementary Table S6. List of *Haemophilus influenzae* strains

Supplementary Table S6. **List of *Haemophilus influenzae* strains**

Organism/Strain	Assembly	Accession_ID
Haemophilus influenzae 1057_HINF	GCA_001053795.1	JWEN01
Haemophilus influenzae 1059_HINF	GCA_001052725.1	JWEL01
Haemophilus influenzae 1061_HINF	GCA_001053815.1	JWEJ01
Haemophilus influenzae 10810	GCA_000210875.1	NC_016809
Haemophilus influenzae 1104	GCA_000636075.1	JFZM01
Haemophilus influenzae 1123_HINF	GCA_001076115.1	JWCI01
Haemophilus influenzae 1124_HINF	GCA_001053885.1	JWCH01
Haemophilus influenzae 1200	GCA_001909005.1	MQMH01
Haemophilus influenzae 1209	GCA_000877255.1	JMQP01
Haemophilus influenzae 177_HINF	GCA_001055615.1	JVRF01
Haemophilus influenzae 2019	GCA_000968335.1	NZ_CP008740
Haemophilus influenzae 22.1-21	GCA_000169735.1	AAZD01
Haemophilus influenzae 22.4-21	GCA_000169855.1	AAZJ01
Haemophilus influenzae 3655	GCA_000169775.1	AAZF01
Haemophilus influenzae 40_HINF	GCA_001056575.1	JVIR01
Haemophilus influenzae 411	GCA_000636055.1	JFZK01
Haemophilus influenzae 477	GCA_000931575.1	NZ_CP007470
Haemophilus influenzae 492_HINF	GCA_001076835.1	JVEZ01
Haemophilus influenzae 536_HINF	GCA_001055275.1	JVDE01
Haemophilus influenzae 552_HINF	GCA_001057195.1	JVCL01
Haemophilus influenzae 584	GCA_000636035.1	JFZL01
Haemophilus influenzae 60294N1	GCA_000818925.1	JXCI01
Haemophilus influenzae 65234 B Hi-2	GCA_001949875.1	MPJG01
Haemophilus influenzae 65234 N Hi-1	GCA_001949825.1	MPJH01
Haemophilus influenzae 723	GCA_000931625.1	NZ_CP007472
Haemophilus influenzae 7P49H1	GCA_000173315.1	ABWV01
Haemophilus influenzae 86-028NP	GCA_000012185.1	NC_007146
Haemophilus influenzae ATCC 10211	GCA_001997355.1	MTGI01
Haemophilus influenzae C10	GCA_001276545.1	LDWA01
Haemophilus influenzae C188	GCA_001909015.1	MQMI01
Haemophilus influenzae C486	GCA_000931605.1	NZ_CP007471
Haemophilus influenzae CCUG 26214	GCA_001679235.1	LZDP01
Haemophilus influenzae CCUG 54503	GCA_001679365.1	LZMU01
Haemophilus influenzae CCUG 60490	GCA_001679415.1	LZMV01
Haemophilus influenzae CGSHiCZ412602	GCA_000698365.1	NZ_CP007805
Haemophilus influenzae DL42	-	U08348.1
Haemophilus influenzae F3031	GCA_000197875.1	NC_014920
Haemophilus influenzae F3047	GCA_000200475.1	NC_014922
Haemophilus influenzae HI1373	GCA_001184695.1	LFDP01
Haemophilus influenzae HI1388	GCA_001184645.1	LFDN01
Haemophilus influenzae HI1394	GCA_001184635.1	LFDM01
Haemophilus influenzae HI1408	GCA_001184705.1	LFDJ01
Haemophilus influenzae HI1413	GCA_001298185.1	LHSM01
Haemophilus influenzae HI1417	GCA_001184615.1	LFDK01
Haemophilus influenzae HI1426	GCA_001184595.1	LFDL01
Haemophilus influenzae HI1722	GCA_001184545.1	LFFU01
Haemophilus influenzae HI1974	GCA_001184535.1	LFFT01
Haemophilus influenzae HI1980	GCA_001184555.1	LFFO01
Haemophilus influenzae HI2004	GCA_001184445.1	LFFQ01
Haemophilus influenzae HI2007	GCA_001184475.1	LFFP01
Haemophilus influenzae HI2114	GCA_001184485.1	LFFR01
Haemophilus influenzae HI2116	GCA_001184515.1	LFFS01
Haemophilus influenzae HI2192	GCA_001298205.1	LHSN01

Haemophilus influenzae HI2428	GCA_001298195.1	LHSO01
Haemophilus influenzae Hi322	GCA_000833835.1	JXLX01
Haemophilus influenzae Hi345	GCA_000833875.1	JXLY01
Haemophilus influenzae Hi359	GCA_000833855.1	JXLZ01
Haemophilus influenzae Hi361	GCA_000833815.1	JXMA01
Haemophilus influenzae Hi375	GCA_000767075.1	NZ_CP009610
Haemophilus influenzae Hi378	GCA_000833895.1	JXMB01
Haemophilus influenzae Hi381	GCA_000833765.1	JXMC01
Haemophilus influenzae Hi394	GCA_000833915.1	JXMD01
Haemophilus influenzae Hi403	GCA_000833935.1	JXME01
Haemophilus influenzae Hi535	GCA_001908955.1	MQMJ01
Haemophilus influenzae KR494	GCA_000465255.1	NC_022356
Haemophilus influenzae MiHi270	GCA_000833755.1	JXMF01
Haemophilus influenzae MiHi64	GCA_000833745.1	JXMG01
Haemophilus influenzae N182	-	U08349.1
Haemophilus influenzae NCTC8143	GCA_001457655.1	NZ_LN831035
Haemophilus influenzae NML-Hia-1	GCA_001856725.1	NZ_CP017811
Haemophilus influenzae NT127	GCA_000175435.1	ACSL01
Haemophilus influenzae PittAA	GCA_000169795.1	AAZG01
Haemophilus influenzae PittEE	GCA_000016465.1	NC_009566
Haemophilus influenzae PittHH	GCA_000169815.1	AAZH01
Haemophilus influenzae PittII	GCA_000169835.1	AAZI01
Haemophilus influenzae R2846	GCA_000165575.1	NC_017452
Haemophilus influenzae R2866	GCA_000165525.1	NC_017451
Haemophilus influenzae R3021	GCA_000169755.1	AAZE01
Haemophilus influenzae Rd KW20	GCA_000027305.1	NC_000907
Haemophilus influenzae RdAW	GCA_000175455.1	ACSM01
Haemophilus influenzae RMHi93	GCA_000833735.1	JXMH01
