# Supplementary material for: The Diversity of Mammalian Hemoproteins and Microbial Heme Scavengers Is Shaped by an Arms Race for Iron Piracy
Source: Front Immunol. 2018 Sep 11;9:2086. doi: 10.3389/fimmu.2018.02086 (PMC6142043; doi:10.3389/fimmu.2018.02086)
Supplement: Supplementary file 7 [file Table_7.PDF]

Supplementary Material

The diversity of mammalian hemoproteins and microbial heme scavengers is shaped by an arms race for iron piracy

Alessandra Mozzi*, Diego Forni, Mario Clerici, Rachele Cagliani, Manuela Sironi

*** Correspondence:** Alessandra Mozzi: alessandra.mozzi@bp.lnf.it

Supplementary Tables

Supplementary Table S7. List of *Pseudomonas aeruginosa* strains

Supplementary Table S7. **List of *Pseudomonas aeruginosa* strains**

**Organism/Strain**	**Assembly**	**Accession ID**
Pseudomonas aeruginosa 12-4-4(59)	GCA_001482325.1	NZ_CP013696.1
Pseudomonas aeruginosa 19BR	GCA_000223945.2	NZ_AFXJ01000001.1
Pseudomonas aeruginosa 213BR	GCA_000223965.2	NZ_AFXK01000001.1
Pseudomonas aeruginosa 39016	GCA_000148745.1	NZ_CM001020.1
Pseudomonas aeruginosa 8380	GCA_001548135.1	NZ_AP014839.1
Pseudomonas aeruginosa AES-1R	GCA_000220025.3	NZ_CP013680.1
Pseudomonas aeruginosa ATCC 15692	GCA_001729505.1	NZ_CP017149.1
Pseudomonas aeruginosa ATCC 27853	GCA_001618925.1	NZ_CP015117.1
Pseudomonas aeruginosa B136-33	GCA_000359505.1	NC_020912.1
Pseudomonas aeruginosa BAMCPA07-48	GCA_001632245.1	NZ_CP015377.1
Pseudomonas aeruginosa C7447m	GCA_000468935.1	NC_022360.1
Pseudomonas aeruginosa Carb01 63	GCA_000981825.1	NZ_CP011317.1
Pseudomonas aeruginosa Cu1510	GCA_001465155.1	CP013144.1
Pseudomonas aeruginosa DHS01	GCA_000496455.2	NZ_CP013993.1
Pseudomonas aeruginosa DK2	GCA_000271365.1	NC_018080.1
Pseudomonas aeruginosa DN1	GCA_001722005.2	NZ_CP017099.1
Pseudomonas aeruginosa DSM 50071	GCA_001045685.1	NZ_CP012001.1
Pseudomonas aeruginosa F22031	GCA_000816985.1	NZ_CP007399.1
Pseudomonas aeruginosa F23197	GCA_001516245.1	NZ_CP008856.1
Pseudomonas aeruginosa F30658	GCA_001516265.1	NZ_CP008857.1
Pseudomonas aeruginosa F63912	GCA_001594325.1	NZ_CP008858.1
Pseudomonas aeruginosa F9670	GCA_001542835.1	NZ_CP008873.1
Pseudomonas aeruginosa F9676	GCA_001077475.1	NZ_CP012066.1
Pseudomonas aeruginosa FA-HZ1	GCA_001750705.1	NZ_CP017353.1
Pseudomonas aeruginosa FRD1	GCA_000829885.1	NZ_CP010555.1
Pseudomonas aeruginosa H27930	GCA_001516325.1	NZ_CP008860.1
Pseudomonas aeruginosa H47921	GCA_001516345.1	NZ_CP008861.1
Pseudomonas aeruginosa H5708	GCA_001516305.1	NZ_CP008859.1
Pseudomonas aeruginosa IOMTU 133	GCA_001548335.1	NZ_AP017302.1
Pseudomonas aeruginosa LES400	GCA_000583935.1	NZ_CP006982.1
Pseudomonas aeruginosa LES431	GCA_000508765.1	NC_023066.1
Pseudomonas aeruginosa LESB58	GCA_000026645.1	NC_011770.1
Pseudomonas aeruginosa LESB65	GCA_000583955.1	NZ_CP006983.1
Pseudomonas aeruginosa LESlike1	GCA_000583975.1	NZ_CP006984.1
Pseudomonas aeruginosa LESlike4	GCA_000583995.1	NZ_CP006985.1
Pseudomonas aeruginosa LESlike5	GCA_000583895.1	NZ_CP006980.1
Pseudomonas aeruginosa LESlike7	GCA_000583915.1	NZ_CP006981.1
Pseudomonas aeruginosa M1608	GCA_001516365.1	NZ_CP008862.1
Pseudomonas aeruginosa M18	GCA_000226155.1	NC_017548.1
Pseudomonas aeruginosa M37351	GCA_001516385.1	NZ_CP008863.1
Pseudomonas aeruginosa MTB-1	GCA_000504045.1	NC_023019.1
Pseudomonas aeruginosa N17-1	GCA_001606045.1	NZ_CP014948.1
Pseudomonas aeruginosa NCGM 1900	GCA_000829275.1	NZ_AP014622.1
Pseudomonas aeruginosa NCGM 1984	GCA_000829255.1	NZ_AP014646.1
Pseudomonas aeruginosa NCGM2.S1	GCA_000284555.1	NC_017549.1
Pseudomonas aeruginosa NCGM257	GCA_001547955.1	NZ_AP014651.1
Pseudomonas aeruginosa NCTC10332	GCA_001457615.1	NZ_LN831024.1
Pseudomonas aeruginosa NHmuc	GCA_001900265.1	NZ_CP013479.1
Pseudomonas aeruginosa PA_D1	GCA_001721745.1	NZ_CP012585.1
Pseudomonas aeruginosa PA_D16	GCA_001721805.1	NZ_CP012581.1
Pseudomonas aeruginosa PA_D2	GCA_001721765.1	NZ_CP012578.1
Pseudomonas aeruginosa PA_D21	GCA_001722045.1	NZ_CP012582.1
Pseudomonas aeruginosa PA_D22	GCA_001721825.1	NZ_CP012583.1

Pseudomonas aeruginosa PA_D25	GCA_001721845.1	NZ_CP012584.1
Pseudomonas aeruginosa PA_D9	GCA_001721785.1	NZ_CP012580.1
Pseudomonas aeruginosa PA1	GCA_000496605.2	NC_022808.2
Pseudomonas aeruginosa PA1088	GCA_001792835.1	NZ_CP015001.1
Pseudomonas aeruginosa PA11803	GCA_001792875.1	NZ_CP015003.1
Pseudomonas aeruginosa PA121617	GCA_001679685.1	NZ_CP016214.1
Pseudomonas aeruginosa PA1R	GCA_000496645.1	NC_022806.1
Pseudomonas aeruginosa PA1RG	GCA_001293085.1	NZ_CP012679.1
Pseudomonas aeruginosa PA38182	GCA_000531435.1	HG530068.1
Pseudomonas aeruginosa PA7	GCA_000017205.1	NC_009656.1
Pseudomonas aeruginosa PA7790	GCA_001870265.1	NZ_CP014999.1
Pseudomonas aeruginosa PA8281	GCA_001792855.1	NZ_CP015002.1
Pseudomonas aeruginosa PA96	GCA_000626655.2	NZ_CP007224.1
Pseudomonas aeruginosa PACS2	GCA_000168335.1	NZ_AAQW01000001.1
Pseudomonas aeruginosa PAER4_119	GCA_001879525.1	NZ_CP013113.1
Pseudomonas aeruginosa PAO1	GCA_000006765.1	NC_002516.2
Pseudomonas aeruginosa PAO1_Orsay	GCA_900070375.1	NZ_LN871187.1
Pseudomonas aeruginosa PAO1-VE13	GCA_000484545.1	NC_022594.1
Pseudomonas aeruginosa PAO1-VE2	GCA_000484495.1	NC_022591.1
Pseudomonas aeruginosa PAO1H2O	GCA_000714515.1	NZ_CP008749.1
Pseudomonas aeruginosa PSE305	GCA_000750905.1	NZ_HG974234.1
Pseudomonas aeruginosa RP73	GCA_000414035.1	NC_021577.1
Pseudomonas aeruginosa S04 90	GCA_000988505.1	NZ_CP011369.1
Pseudomonas aeruginosa S86968	GCA_001515845.1	NZ_CP008865.1
Pseudomonas aeruginosa SCV20265	GCA_000510305.1	NC_023149.1
Pseudomonas aeruginosa SCVFeb	GCA_001900195.1	NZ_CP013477.1
Pseudomonas aeruginosa SCVJan	GCA_001900225.1	NZ_CP013478.1
Pseudomonas aeruginosa SJTD-1	GCA_000271985.2	NZ_CP015877.1
Pseudomonas aeruginosa T38079	GCA_001515915.1	NZ_CP008866.1
Pseudomonas aeruginosa T52373	GCA_001516005.1	NZ_CP008867.1
Pseudomonas aeruginosa T63266	GCA_001516105.1	NZ_CP008868.1
Pseudomonas aeruginosa UCBPP-PA14	GCA_000014625.1	NC_008463.1
Pseudomonas aeruginosa USDA-ARS-USMARC-41639	GCA_001518975.1	NZ_CP013989.1
Pseudomonas aeruginosa VA-134	GCA_001447845.1	NZ_CP013245.1
Pseudomonas aeruginosa VRFPA04	GCA_000473745.3	NZ_CP008739.1
Pseudomonas aeruginosa W16407	GCA_001516165.1	NZ_CP008869.1
Pseudomonas aeruginosa W36662	GCA_001516185.1	NZ_CP008870.1
Pseudomonas aeruginosa W45909	GCA_001516205.1	NZ_CP008871.1
Pseudomonas aeruginosa W60856	GCA_001516225.1	NZ_CP008864.1
Pseudomonas aeruginosa X78812	GCA_001542795.1	NZ_CP008872.1
Pseudomonas aeruginosa YL84	GCA_000524595.1	NZ_CP007147.1
