# Supplementary material for: The Diversity of Mammalian Hemoproteins and Microbial Heme Scavengers Is Shaped by an Arms Race for Iron Piracy
Source: Front Immunol. 2018 Sep 11;9:2086. doi: 10.3389/fimmu.2018.02086 (PMC6142043; doi:10.3389/fimmu.2018.02086)
Supplement: Supplementary file 8 [file Table_8.PDF]

Supplementary Material

The diversity of mammalian hemoproteins and microbial heme scavengers is shaped by an arms race for iron piracy

Alessandra Mozzi*, Diego Forni, Mario Clerici, Rachele Cagliani, Manuela Sironi

*** Correspondence:** Alessandra Mozzi: alessandra.mozzi@bp.lnf.it

Supplementary Tables

Supplementary Table S8. Prior distribution used for omegaMap analysis

Supplementary Table S8. Prior distribution used for omegaMap analysis.

Parameter	Prior
μ (synonymous transversion)	Exponential (mean 0.07)
κ (transition/transversion rate)	Exponential (mean 3.0)
ϕ (insertion/deletion rate)	Exponential (mean 0.1)
ω (dN/dS)	Exponential (mean 1.0)
ρ (recombination)	Exponential (mean 0.1)
omegaBlok	10
rhoBlock	30
Thinning	100
Iterations	1000000
Burn-in	50000
