# Supplementary material for: The Diversity of Mammalian Hemoproteins and Microbial Heme Scavengers Is Shaped by an Arms Race for Iron Piracy
Source: Front Immunol. 2018 Sep 11;9:2086. doi: 10.3389/fimmu.2018.02086 (PMC6142043; doi:10.3389/fimmu.2018.02086)
Supplement: Supplementary file 9 [file Table_9.PDF]

Supplementary Material

The diversity of mammalian hemoproteins and microbial heme scavengers is shaped by an arms race for iron piracy

Alessandra Mozzi*, Diego Forni, Mario Clerici, Rachele Cagliani, Manuela Sironi

*** Correspondence:** Alessandra Mozzi: alessandra.mozzi@bp.lnf.it

Supplementary Tables

Supplementary Table S9. Substitution saturation analysis

Supplementary Table S9. Substitution saturation analysis

Gene	I_{ss}[a]	I_{ssCsym}[b]	Degrees of freedom	*p* value[c]
HBB	0.173	0.683	235	<0.0001
HPX	0.210	0.735	748	<0.0001

Notes:

a. Index of substitution saturation

b. Critical value for a symmetrical tree topology

c. Probability that I_{ss} is significantly different from the critical value
