# Supplementary material for: The Diversity of Mammalian Hemoproteins and Microbial Heme Scavengers Is Shaped by an Arms Race for Iron Piracy
Source: Front Immunol. 2018 Sep 11;9:2086. doi: 10.3389/fimmu.2018.02086 (PMC6142043; doi:10.3389/fimmu.2018.02086)
Supplement: Supplementary file 10 [file Table_10.PDF]

Supplementary Material

The diversity of mammalian hemoproteins and microbial heme scavengers is shaped by an arms race for iron piracy

Alessandra Mozzi*, Diego Forni, Mario Clerici, Rachele Cagliani, Manuela Sironi

*** Correspondence:** Alessandra Mozzi: alessandra.mozzi@bp.lnf.it

Supplementary Tables

Supplementary Table S10. SLAC values in mammalian phylogenies

Supplementary Table S10. SLAC values in mammalian phylogenies

Gene	N species	Tree length (Substitutions/Site)	mean dN/dS	Confidence Interval (95%)
HBB	50	1.719	0.339	0.302 – 0.378
HPX	102	4.452	0.439	0.423 – 0.456
