# Supplementary material for: The Diversity of Mammalian Hemoproteins and Microbial Heme Scavengers Is Shaped by an Arms Race for Iron Piracy
Source: Front Immunol. 2018 Sep 11;9:2086. doi: 10.3389/fimmu.2018.02086 (PMC6142043; doi:10.3389/fimmu.2018.02086)
Supplement: Supplementary file 11 [file Table_11.PDF]

Supplementary Material

The diversity of mammalian hemoproteins and microbial heme scavengers is shaped by an arms race for iron piracy

Alessandra Mozzi*, Diego Forni, Mario Clerici, Rachele Cagliani, Manuela Sironi

*** Correspondence:** Alessandra Mozzi: alessandra.mozzi@bp.lnf.it

Supplementary Tables

Supplementary Table S11. Likelihood ratio test (LRT) statistics for models of variable selective pressure among sites in mammalian phylogenies

Supplementary Table S11. Likelihood ratio test (LRT) statistics for models of variable selective pressure among sites in mammalian phylogenies.

Gene	Codon frequency	Selection Model[a]	Degrees of freedom	−2ΔlnL[b]	*p* value	Positively selected sites[c]
HBB	F3x4	M1a vs M2a	2	12.804	1.66×10^{-3}	A14, T51, D53
		M7 vs M8	2	16.833	2.21×10^{-4}	
		M8a vs M8	1	10.080	1.50×10^{-3}	
	F61	M1a vs M2a	2	20.112	4.28×10^{-5}	
		M7 vs M8	2	26.573	1.70×10^{-6}	
		M8a vs M8	1	19.728	8.93×10^{-6}	
HPX	F3x4	M1a vs M2a	2	103.944	2.68×10^{-23}	A7, V9, S78, D82, N106, A156, L172, G175, M177, S181, R222, G243, P252, M255, A298, S359, I365, A447, T461
		M7 vs M8	2	112.158	2.42×10^{-25}	
		M8a vs M8	1	76.933	1.77×10^{-18}	
	F61	M1a vs M2a	2	86.169	1.94×10^{-19}	
		M7 vs M8	2	95.741	1.62×10^{-21}	
		M8a vs M8	1	64.380	1.03×10^{-15}	

Notes:
a. M1a is a nearly neutral model that assumes one ω class between 0 and 1 and one class with ω=1; M2a (positive selection model) is the same as M1a plus an extra class of ω>1; M7 is a null model that assumes that 0<ω<1 is beta distributed among sites;
M8 (positive selection model) is the same as M7 but also includes an extra category of sites with ω>1.
M8a is the same as M8, except that the 11th category cannot allow positive selection, but only neutral evolution.
b. 2ΔlnL: twice the difference of the natural logs of the maximum likelihood of the models being compared.
c. Positions refer to the human sequence.
