# Supplementary material for: The Diversity of Mammalian Hemoproteins and Microbial Heme Scavengers Is Shaped by an Arms Race for Iron Piracy
Source: Front Immunol. 2018 Sep 11;9:2086. doi: 10.3389/fimmu.2018.02086 (PMC6142043; doi:10.3389/fimmu.2018.02086)
Supplement: Supplementary file 12 [file Table_12.PDF]

Supplementary Material

The diversity of mammalian hemoproteins and microbial heme scavengers is shaped by an arms race for iron piracy

Alessandra Mozzi*, Diego Forni, Mario Clerici, Rachele Cagliani, Manuela Sironi

*** Correspondence:** Alessandra Mozzi: alessandra.mozzi@bp.lnf.it

Supplementary Tables

Supplementary Table S12. Tests of episodic positive selection among branches

Supplementary Table S12. Tests of episodic positive selection among branches.

Gene<br>Foreground branch	**Model**	**−2ΔlnL**[b]	***p* value (FDR corrected)**	**Positively selected sites**[c]
HBB				
Caprinae	MA1 vs MA[a]	12.243	4.67×10^{-4}	S45
HPX				
Great roundleaf bat	MA1 vs MA[a]	12.160	4.88×10^{-4} (7.08×10^{-4})	--
Common bottlenose dolphin	MA1 vs MA[a]	11.467	7.08×10^{-4} (7.08×10^{-4})	--

Notes:
a. MA and MA1 are branch-site models that assume four classes of sites: the MA model allows a proportion of codons to have ω≥1 on the foreground branches, whereas the MA1 model does not.
b. 2ΔlnL: twice the difference of the natural logs of the maximum likelihood of the models being compared.
c. Positions refer to the human sequence.
