# Supplementary material for: The Diversity of Mammalian Hemoproteins and Microbial Heme Scavengers Is Shaped by an Arms Race for Iron Piracy
Source: Front Immunol. 2018 Sep 11;9:2086. doi: 10.3389/fimmu.2018.02086 (PMC6142043; doi:10.3389/fimmu.2018.02086)
Supplement: Supplementary file 14 [file Table_14.PDF]

Supplementary Material

The diversity of mammalian hemoproteins and microbial heme scavengers is shaped by an arms race for iron piracy

Alessandra Mozzi*, Diego Forni, Mario Clerici, Rachele Cagliani, Manuela Sironi

*** Correspondence:** Alessandra Mozzi: alessandra.mozzi@bp.lnf.it

Supplementary Tables

Supplementary Table S14. Homology model reports by SWISS-MODEL server

Supplementary Table S14. Homology model reports by SWISS-MODEL server

Target	Template	Sequence identity	Sequence similarity	Coverage	GMQE	QMEAN
*Nm*HpuA	5EE4_A	33.80	0.37	0.84	0.62	-2.32
*Hg*Hx	4RT6_B	85.12	0.59	0.88	0.82	-1.45
