# Supplementary material for: The Diversity of Mammalian Hemoproteins and Microbial Heme Scavengers Is Shaped by an Arms Race for Iron Piracy
Source: Front Immunol. 2018 Sep 11;9:2086. doi: 10.3389/fimmu.2018.02086 (PMC6142043; doi:10.3389/fimmu.2018.02086)
Supplement: Supplementary file 15 [file Table_15.PDF]

Supplementary Material

The diversity of mammalian hemoproteins and microbial heme scavengers is shaped by an arms race for iron piracy

Alessandra Mozzi*, Diego Forni, Mario Clerici, Rachele Cagliani, Manuela Sironi

*** Correspondence:** Alessandra Mozzi: alessandra.mozzi@bp.lnf.it

Supplementary Tables

Supplementary Table S15. Tertiary structure report by Raptor X server

Supplementary Table S15. Tertiary structure report by Raptor X server

Target	Template	Modeled residues	Disorderd positions	uGDT	(GDT)	*p* value
*Nm*HpuB	3v89_A	810 (100%)	44 (5%)	377	46	1.50×10^{-25}
